# Recent Advances in Ionic Liquids in Biomedicine

**DOI:** 10.1002/advs.202004819

**Published:** 2021-07-10

**Authors:** Alexander M. Curreri, Samir Mitragotri, Eden E. L. Tanner

**Affiliations:** ^1^ John A. Paulson School of Engineering and Applied Sciences Harvard University Cambridge MA 02138 USA; ^2^ Wyss Institute of Biologically Inspired Engineering Boston MA 02115 USA; ^3^ Present address: Department of Chemistry and Biochemistry The University of Mississippi University MS 38677 USA

**Keywords:** deep eutectic solvents, drug delivery, ionic liquids, ionic solvents

## Abstract

The use of ionic liquids and deep eutectic solvents in biomedical applications has grown dramatically in recent years due to their unique properties and their inherent tunability. This review will introduce ionic liquids and deep eutectics and discuss their biomedical applications, namely solubilization of drugs, creation of active pharmaceutical ingredients, delivery of pharmaceuticals through biological barriers, stabilization of proteins and other nucleic acids, antibacterial agents, and development of new biosensors. Current challenges and future outlooks are discussed, including biocompatibility, the potential impact of the presence of impurities, and the importance of understanding the microscopic interactions in ionic liquids in order to design task‐specific solvents.

## Graphical abstract

This review covers historical and recent advances of ionic liquids within the field of biomedicine. The article focuses on solubilization of drugs, active pharmaceutical ingredient ionic liquids, drug delivery, nanocarriers, protein stabilization, antimicrobial agents, and biosensing. The review also provides the future outlook and challenges for ionic liquids in the field of biomedical science.

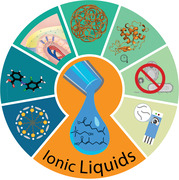

## 1 Introduction

Ionic liquids (ILs) are defined as solvents that consist of bulky and asymmetric organic anions and cations that exist as liquids below 100 °C. Deep eutectic solvents (DES) are defined as materials that, once combined, experience a depression in the melting point due predominantly to the formation of hydrogen bonds.^[^ ^1^ ^]^ They typically consist of non‐binary mixtures of anions and cations, whereas ionic liquids classically include a 1:1 combination that is dominated by non‐directional ionic interactions. Many may exist along a continuum between these two.^[^ ^2^ ^]^

**Figure 1:**
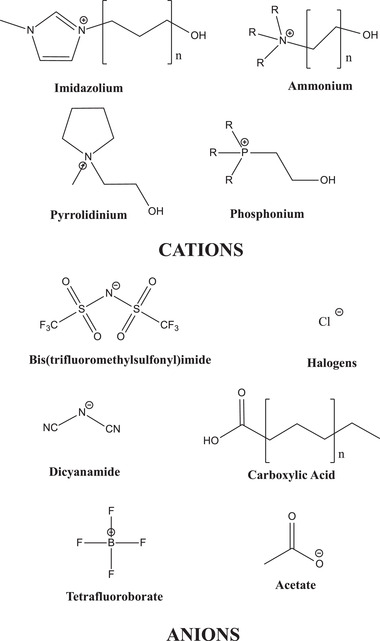
Common cations and anions used to create ionic liquids.

Ionic liquids and deep eutectic solvents have a number of properties that make them desirable for use in biomedical applications. The selection of ions has a strong impact on the physico‐chemical properties and biological outcome of the ionic liquids. **Figure** **1** shows common cations and anions that are used to create ionic liquids.

Perhaps the most critical ionic liquid characteristic, relevant to their use in biomedicine, is their solubility in both polar and nonpolar solvents. This will be discussed in greater detail below, as there are a number of fundamental chemical details that allow for its unique miscibility. Melting temperature is also an extremely important property, as it is often used as a descriptor for what qualifies as an ionic liquid, whereby ionic liquids are defined as having melting points below 100 °C.^[^ ^3^ ^]^ The reason ionic liquids have substantially lower melting temperatures than standard ionic compounds, like NaCl, is a result of their inability to pack into a well‐defined crystal structure.^[^ ^4^ ^]^ This is due to the bulky nature of the ions involved and is attributed to three major factors: intermolecular forces between the ions involved in the solvent, ion symmetry, and ion conformational degrees of freedom.^[^ ^5^ ^]^ Weaker intermolecular forces including ionic and hydrogen bonding interactions reduce the tightness of packing.^[^ ^6^ ^]^ Ion symmetry works in a similar way because asymmetrical compounds cannot pack easily into repeating lattice structures.^[^ ^7^ ^]^ Conformational degrees of freedom work to prevent crystal lattice symmetry and formation of consistent intermolecular interactions.^[^ ^8^ ^]^ Researchers can control these properties to decrease the melting point of ionic liquids by utilizing ions that allow for delocalization of charges, those that are asymmetrical in nature, and those that have long substituents with many rotational degrees of freedom.^[^ ^5^ ^]^ In biomedical applications, ionic liquids often have melting temperatures below room temperature, so called room‐temperature ILs (RTILs), as they need to be used in a liquid form at physiologically relevant temperatures (≈37 °C).

The liquid nature at these relevant temperatures gives ionic liquids their inherent conductivity. Unlike ionic solids, many ionic liquids are molten at body temperature and do not need to be dissolved in other solvents to conduct electricity. Because ions are free to move in the liquid state, they easily allow for transfer of electrons through the bulk compound. Thus, conductivity can be controlled by manipulating ion size and charge. Use of smaller cations and anions allows for easier ion mobility, which corresponds to higher conductivities. Additionally, use of highly charged molecules prevents ion mobility due to the occurrence of more ion‐ion interactions, which leads to lower conductivity.^[^ ^9^ ^]^ This impact of charge mobility on conductivity appears to apply more to cations than anions.^[^ ^10^ ^]^ These intermolecular interactions that can prevent ion mobility give rise to another relevant property, viscosity. Unsurprisingly, like other fluids, the viscosity of ionic liquids is mostly mediated by intermolecular forces.^[^ ^11^ ^]^ These can range in strength from ion–ion interactions down to dispersion forces, with hydrogen bonding playing a key role in biocompatible ionic liquids. Strong intermolecular forces, like ion–ion and dipole–dipole, prevent ionic liquid layers from being able to easily shear past each other, but even dispersion forces can drive viscosity. This causes many RTILs to possess viscosities in the hundreds to thousands of centipoise.^[^ ^12^, ^13^ ^]^ Viscosity can be increased or decreased by selective use of molecules that inherently possess greater intermolecular forces. For example, within a certain class of ionic liquids, say ammonium‐based, utilizing anions with longer alkyl chains can allow for stronger dispersion forces and increase viscosity. This phenomenon is similar to how the viscosity of hydrocarbons increase viscosity with increased chain length.^[^ ^14^ ^]^

**Figure 2:**
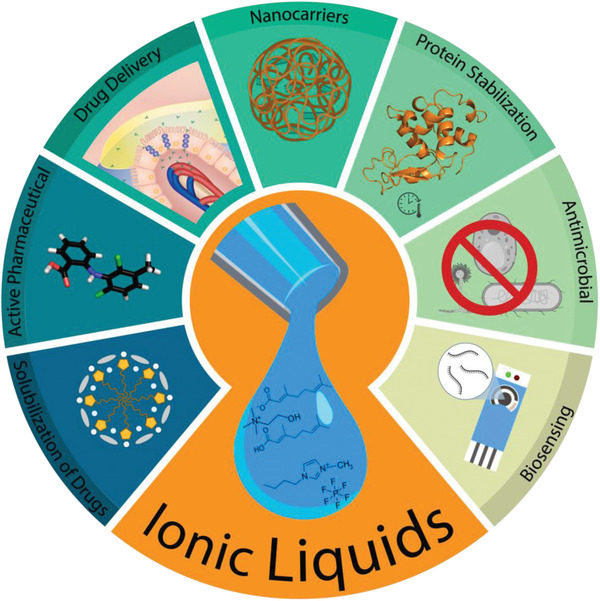
Biomedical applications of ionic liquids.

Perhaps one of the most important properties that differentiates the use of biorelevant ionic liquids from ones used in traditional chemistry applications is their miscibility, especially with water. Whether or not an ionic liquid will dissolve in water is entirely dependent upon the solvation energy and whether it is sufficient to overcome the ion cohesive forces. If the free energy is lower in the dissolved state than the biphasic state, then the ionic liquid will be miscible. The free energy is also dependent upon the “hydrophobic effect,” which comes as a significant free energy penalty because it prevents water molecules from forming normal tetrahedral hydrogen bonding geometries. It should be noted that miscibility, as well as conductivity and viscosity, have a significant dependence on temperature and so the properties can change from normal room temperature lab conditions to physiological temperatures.^[^ ^15^ ^]^ Additionally, some biorelevant ILs are miscible in hydrophobic solvents as well as water. These amphiphilic ionic liquids generally have hydrophobic substituents on one end of a longer chain and hydrophilic substituents on the other end.^[^ ^16^ ^]^ Miscibility, and in turn amphiphilicity, can be tuned by incorporating long hydrocarbon chains or aromatic rings for poorer water solubility or carboxyl, alcohol groups, and nitrogen atoms to increase hydrophilicity through hydrogen bonding. The combination of the above properties has led to extensive use of ILs in biomedicine. Both their liquid state and miscibility, in polar and non‐polar solvents, aids in solubilization of hydrophobic drugs in aqueous formulations and penetration of hydrophobic barriers in the body. Their inherent conductivity allows for easy charge transfer in biosensors. The field of biocompatible ILs has now expanded its horizons to include a wider range of functions including protein stabilization, active pharmaceutical ingredient development, drug delivery of macromolecules, antimicrobial agents, modification of nanocarriers, and in biosensing.^[^ ^17^ ^]^ **Figure** **2** depicts representations of the applications of ILs in each of these fields and Table S1, Supporting Information, provides a summary of the uses with those fields. This review focuses on recent developments of ILs in these fields, provides thoughts on some of the shortcomings facing biocompatible IL research, and discusses what the future of ILs for biomedical use might look like.

### 1.1 A Brief History of Ionic Liquids and Deep Eutectic Solvents

The first ionic liquid, ethylammonium nitrate, was made by Paul Walden in 1914.^[^ ^18^ ^]^ This first ionic liquid was not used widely due to its air and moisture sensitivity, which made its handling difficult. This changed in 1992 when the first set of air‐ and moisture‐stable ILs, based on the imidazolium cation, were reported by Wilkes and Zaworotko.^[^ ^19^ ^]^ Since then, ILs have been broadly used across Chemistry, Engineering, and Materials Science owing to a slew of favorable properties including low volatility, high thermal and electrochemical stability, recyclability and, most importantly, tunability. The tunability arises from the fact that changing the atomistic components of the IL results in shifts in the properties of the solvent, such as viscosity, conductivity, and miscibility with other solvents, including water. Deep eutectic solvents (DES) were first reported in 2001, and grew in popularity because of their ease of synthesis, and the fact that they were largely composed of inexpensive and often biocompatible materials.

The use of ionic liquids for biomedical applications started in the late 1990s and early 2000s where they were used to enhance thermostability of both enzymes^[^ ^20^ ^]^ and model proteins^[^ ^21^ ^]^ as well as augment enzymatic catalytic efficiency.^[^ ^22^ ^]^ Additionally, the 2000s saw the use of ILs as antimicrobial agents^[^ ^23^ ^]^ and for anti‐cancer drug synthesis.^[^ ^24^ ^]^ ILs were also used in controlled release systems^[^ ^25^ ^]^ and as formulation excipients^[^ ^26^ ^]^ for poorly water soluble small molecules. Over the years, ILs have expanded into these fields largely as a result of their fundamental chemical properties that make them unique biomedically effective compounds.

## 2 Biomedical Applications

### 2.1 Solubilization of Drugs

Many molecules employed as pharmaceutical agents have very poor solubility in water and biological media, thus substantially limiting their therapeutic utility. Solubilization is controlled by three major events; the disruption of the interactions within the solute lattice, the formation of “holes” in the solvent, and the interaction and integration of the solute into the “hole” formed in the solvent.^[^ ^27^ ^]^ Ionic liquids have been used to successfully solubilize a range of poorly soluble compounds, primarily through their ability to interact with the solute and, if present, co‐solvent molecules, disrupting the existing interactions and promoting integration of the solute. Many of the early IL drug solubilization studies focused on quantifying solubility of small molecule drugs in neat and aqueous IL formulations.^[^ ^28^ ^]^ These early studies particularly focused on imidazolium and quaternary ammonium ILs.^[^ ^29^ ^]^ Work in the early 2010s started to provide insights into the supramolecular structure of the IL‐drug formulations,^[^ ^30^ ^]^ solubility parameters,^[^ ^31^ ^]^ and broadened the scope to include phosphonium and pyrrolidinium cations.^[^ ^31^ ^]^ The mid‐2010s saw the development of a number of specifically tuned ILs^[^ ^32^ ^]^ and in vitro assessments of retention of drug efficacy.^[^ ^33^ ^]^ Recently, Lofti et al.^[^ ^34^ ^]^ used a conductor like screening model for real solvents to predict the solubility of an antiviral, acyclovir, in neat ILs, and experimentally validated choline‐acetate systems as providing the highest solubility. Work out of the same lab was not only able to improve upon the solubility of acyclovir using cholinium amino acid ILs but also allowed for transdermal delivery of the drug.^[^ ^35^ ^]^ The use of ammonium and phosphonium chlorides showed enhancements of 60‐ to 120‐fold over aqueous solutions alone, and the use of imidazolium thiocyanate and dicyanamide demonstrated that both the cation and anion can operate synergistically to mediate interactions between the organic molecule and the aqueous solution, thus increasing the overall solubility.^[^ ^36^ ^]^ Cholinium amino acid ILs were used to solubilize and stabilize paclitaxel, sterically and ionically, preventing the drug molecules from aggregating for 3 months.^[^ ^37^ ^]^ Farooq et al.^[^ ^38^ ^]^ evidenced enhanced solubility through the formation of micelles by a surface active imidazolium chloride IL, with the antidepressant nortriptyline hydrochloride being trapped at the inner surface of the microstructures. Pal and Yadav^[^ ^39^ ^]^ used a similar IL with a higher degree of substitution to solubilize anesthetics lidocaine hydrochloride and procaine hydrochloride, evidencing strong interactions between the drug molecules and the IL components. Chowdhury and coworkers utilized a choline oleic acid IL to form complexes with curcumin, increasing the aqueous solubility from 30 nmol to ≈22 mmol.^[^ ^40^ ^]^ The same choline oleic IL was used to form microemulsions that increased solubility of celecoxib, acyclovir, methotrexate, and dantrolene sodium by greater than 20 times in water.^[^ ^41^ ^]^ Mirheydari et al.^[^ ^42^ ^]^ investigated the anticonvulsant Lamotrigine, reporting a three order of magnitude increase in solubility using an imidazolium bromide IL. Interestingly, the maximum solubility was obtained at a 0.8 mass fraction of the IL in an aqueous solution. The increased solubility upon increasing the mass fraction of the IL up to 0.8 was shown to originate from ion‐polar and dipolar interactions between the IL moieties and the drug molecules. The decrease in solubility past that threshold was attributed to the increased viscosity of the overall formulation and the lower mobility of the IL components, which limits their potential interactions with the drug molecules. As the IL‐drug solubilization field advances, developing the knowledge about the interactions that drive solubilization is key. Molecular dynamics simulations have been used to analyze the interaction between LASSBio‐294, a cardiovascular drug, and ILs, allowing for a higher throughput screen method than wet lab experiments can allow.^[^ ^43^ ^]^ A summary of the publications covered in this section can be found in Table S2, Supporting Information. Overall, a variety of ILs are being used to substantially increase the aqueous solubility of otherwise sparingly soluble pharmaceutical candidates, and this process is largely driven by the ILs interacting with the drug molecules. Most of the work discussed here focuses on formulation and characterization of physicochemical properties, with some work being undertaken to establish in vitro toxicity. Comprehensive studies are still required to establish what effect, if any, the presence of IL in the formulations will have in vivo and if the IL‐drug interactions that result in the enhanced solubility will affect drug efficacy. This should be further assessed by understanding the interplay of interactions between ILs, drugs, and the targets of interest. ILs in solution should not be thought of as bulk electrolytes because they can form structures on the nanometer and micrometer level. This will be discussed further in the Section 2.4, but researchers can perform molecular dynamics assessments of these interactions in single‐drug IL aqueous solutions in order to advance knowledge in the realm of IL drug solubilization.

### 2.2 Active Pharmaceutical Ingredient‐Ionic Liquid (API‐IL)

Challenges of poor solubility, thermal stability, and bioavailability of traditional pharmaceutics have also been addressed by transforming the drug molecules themselves into ionic liquids—referred to as Active Pharmaceutical Ingredient‐Ionic Liquids (API‐ILs). The logic behind API‐ILs is that the profile of the drug can be maintained while imbuing it with the desirable properties of the counter‐ion, and of the solvent class as a whole. The first wave of API‐ILs were largely focused on synthesis of these new liquid salts to incorporate well known pharmacophores.^[^ ^44^, ^45^, ^46^ ^]^ Research in the early 2010s began to shift toward evaluating API‐IL characterization and starting to assess formulation physiochemical characteristics,^[^ ^47^ ^]^ in vitro antibacterial efficacy,^[^ ^48^ ^]^ and potential for delayed release.^[^ ^49^ ^]^ Recent literature is heavily focused on the synthesis and characterization of API‐ILs. Ibuprofen was paired with a benzylammonium cation and investigated theoretically with density functional theory and experimentally with Raman and infrared spectroscopy, with the authors reporting that the ibuprofen‐IL possessed greater stabilization than the non‐IL form, due to the delocalization of charge on the cation.^[^ ^50^ ^]^ A benzethonium proline API‐IL was prepared and characterized by Yan et al.^[^ ^51^ ^]^ in ternary mixtures containing water and small biomolecules. The properties of the mixtures are governed by the interactions of the ionic components with the hydrophilic components of the biomolecules, not unlike the IL‐facilitated solubilization discussed in the previous section. Similarly, an imidazolium salicylate IL was synthesized and characterized in the presence of glycine and L‐alanine, where the authors indicated that the thermodynamics of the solution was also dominated by the interactions between the ions and the polar domains of the amino acids.^[^ ^52^ ^]^

**Figure 3:**
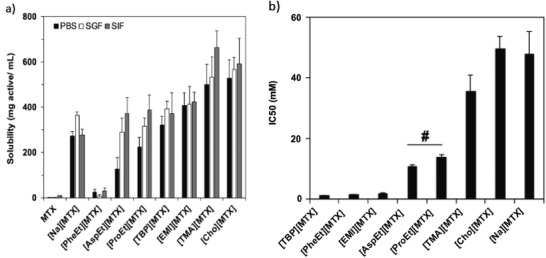
a) MTX‐ILs solubilities in in various buffers are comparable or better solubilities to MTX and [Na][MTX]. b) In vitro antitumor activity, as measured by HeLa cell viability, shows MTX‐ILs have significantly lower IC50 values than the standard treatment of [Na][MTX]. Reproduced with permission.^[^ ^55^ ^]^ Copyright 2019, Elsevier.

In terms of the effect of API‐ILs on aqueous solubility, Carvedilol, a beta and alpha blocker used in the management of cardiovascular conditions, was prepared with three bio‐compatible anions containing carbonyl groups: citric, tartaric and saccharin. All three API‐ILs showed doubling of the solubility compared to the free drug.^[^ ^53^ ^]^ Halayqa et al.^[^ ^54^ ^]^ also showed enhanced aqueous solubility of mefenamic acid when synthesized as an ammonium‐IL. An API‐IL of methotrexate, a chemotherapeutic, was prepared with a choline counter‐ion, showing a 5000‐fold increase in aqueous solubility and lower IC_50_ values against a cancer cell line, as depicted in **Figure** **3**.^[^ ^55^ ^]^ Silva et al.^[^ ^56^ ^]^ synthesized a diclofenac‐imidazolium ionic liquid, which has a melting point around 92 °C, but showed 100 times improvement in drug solubility in water. They found that the IL formation was driven largely by transfer of the hydrogen atom from the carboxylic acid to the imidazolium ring nitrogen.

In addition to physicochemical characterization, some work has been undertaken to assess the API‐ILs in a biomedical context, particularly with respect to cytotoxicity and transport across biological membranes. Recently, alendronic acid (ALN), which has previously shown efficacy in postmenopausal osteoporosis, malignant hypercalcemia, and Paget's disease, has been incorporated as a new API‐IL anion. Using a variety of cations, the researchers tested ALN ILs on a variety of cancer cell lines. [C_2_OHMIM][ALN] had similar IC_50_ values to paclitaxel in lung and osteosarcoma cancer cell lines, but was 100 000 less toxic to healthy human cells.^[^ ^57^ ^]^

Amino acid salicylate ILs were prepared and shown to possess lower cytotoxicity and ninefold greater transport across the skin compared with the control sodium‐salicylate.^[^ ^58^ ^]^ Imidazolium salicylate ILs cured with photo‐crosslinking have allowed for the invention of a simple acne patch for effective treatment in an mouse acne model.^[^ ^59^ ^]^ Other small molecule drugs have also been incorporated into API‐ILs for topical treatments. Berton and co‐workers^[^ ^60^ ^]^ compared lidocaine chloride to lidocaine ibuprofen and lidocaine docusate APIs applied topically in vivo in rats. They found that the greatest systemic adsorption occurred using the lidocaine ibuprofen, with minimal amounts of lidocaine found in the blood plasma following treatment with lidocaine docusate, indicating the importance of selection of counter‐ion. More recently, lidocaine ibuprofen has been incorporated into zein filaments, using extrusion, to allow for a sustained release of the API‐IL. In physiologically relevant conditions the IL‐zein filaments showed sustained release of ibuprofen and lidocaine for two weeks, a drastic improvement over the non‐extruded materials which had released both drugs after 60 h.^[^ ^61^ ^]^ Ren et al.^[^ ^62^ ^]^ also prepared Ibuprofen API‐ILs, based on ammonium and phosphonium cations, and evidenced a fivefold enhancement over free ibuprofen in vitro. Sahbaz et al.^[^ ^63^ ^]^ prepared a range of API‐ILs composed of weakly acidic organic anions and lipophilic, mostly ammonium‐based, cations. They investigated oral in vivo adsorption in a rat model, finding that, when administered at the same concentration, the API‐ILs and free drugs showed similar absorption profiles. However, at higher concentrations of the API‐ILs, slower absorption over a longer time period was observed. This was attributed by the authors to reduced partitioning of the IL components into the gastric fluid. This partitioning may pose more of a problem when using API‐ILs compared with free drug solubilized in an IL, and points to the need for comprehensive pharmacokinetic profiling of API‐ILs. A summary of papers covered in this section can be found in Table S3, Supporting Information. In all, transforming therapeutics into ILs is a promising route to improving their aqueous solubility, bioavailability, and toxicity. Further consideration on the systemic impact and pharmacokinetics of API‐ILs is required. In particular research into the molecular pharmacokinetics, an assessment of API‐IL interaction with cell membranes and extracellular space, would allow for better design as the number of API that are being incorporated grows.

### 2.3 Drug Delivery

Once a satisfactory drug formulation has been prepared, delivery poses the next challenge. The human body possesses complex defense systems to prevent the entry of toxins and pathogens, which also restricts the effective delivery of drugs. Ionic liquids have shown great promise in navigating biological barriers such as cell membranes^[^ ^64^ ^]^ and have been particularly effective at penetrating skin.^[^ ^26^, ^65^, ^66^, ^67^ ^]^ In addition, their ability for controlled release from biphasic mixtures,^[^ ^25^ ^]^ ionogels,^[^ ^68^ ^]^ and thermoresponsive gels^[^ ^69^ ^]^ have enabled their incorporation into a variety of drug delivery vehicles. In particular, a variety of ILs have been utilized to form micro and nanoemulsions. In terms of synthesis and characterization, Sanchez‐Fernandez et al.^[^ ^70^ ^]^ investigated the formation of micelles when an ammonium bromide surfactant was added to choline malonic‐based DES with varying amounts of water. The morphology of the micelles changed with the chain length of the surfactant and with the amount of water present due to shifts in the interactions between the surfactant headgroup and the ionic liquid, although the precise nature of these interactions remains to be studied. Pyne et al.^[^ ^71^ ^]^ utilized a cholesterol‐based surface active IL to create and characterize IL‐in‐oil emulsions and vesicles that form spontaneously in water. Poh et al.^[^ ^72^ ^]^ also synthesized a stable imidazolium acetate‐in‐oil emulsion. In terms of drug loading, Kandasamy et al.^[^ ^73^ ^]^ manufactured imidazolium and ammonium acetate IL‐in‐oil emulsions and demonstrated that ammonium‐based microemulsions showed superior loading of acyclovir and methotrexate over imidazolium‐based formulations. Vashishat et al.^[^ ^74^ ^]^ utilized a surface‐active imidazolium dodecylsulfate IL to form microemulsions in water that encapsulated anesthetic tetracaine. Roy et al.^[^ ^75^ ^]^ encapsulated vitamin E in a long chained imidazolium chloride and a surfactant that destabilizes to release the vitamin E molecule in the presence of bile salt. Sastry et al.^[^ ^76^ ^]^ reported the incorporation of sparingly soluble steroid dexamethasone in a similar surface active IL, evidencing increased solubility and extended release from the IL microemulsion. Extended release of chemotherapeutic camptothecin was also reported using a choline chloride/citric acid DES based micelle.^[^ ^77^ ^]^ Two different imidazolium ILs were combined with surfactant Tween to form nanoemulsions that enclosed analgesic piroxicam and showed favorable in vitro release.^[^ ^78^ ^]^ Esson et al. created amphotericin B, an antifungal drug, nanoemulsion using dicholinium ionic liquid‐in‐water that prevented aggregation of the drug and increased solubility. Since the aggregated form can cause cell death in humans, this new formulation could allow for IV administration of the drug. In vitro assays showed no hemolysis above 10 ug mL^−1^, while the free drug caused 100% hemolysis.^[^ ^79^ ^]^ An imidazolium dodecylsulfate IL was used to incorporate hemostatic dencichine into a nano‐carrier that was characterized and tested for transdermal delivery, showing a tenfold transport enhancement over the control.^[^ ^80^ ^]^

The ability of ionic liquids to solubilize polymers, especially naturally occurring cellulose derivatives, and facilitate uniform regeneration has led to their wide use in the synthesis of polymer‐based drug delivery systems. Du et al.^[^ ^81^ ^]^ report the polymerization of a cellulose‐based polymer solvated in an imidazolium chloride that showed pH responsive release of aspirin. Starch/Cellulose gels designed for use as gastric delivery systems were synthesized by dissolving and regenerating the starting materials in imidazolium acetate ILs.^[^ ^82^ ^]^ Bielas et al.^[^ ^83^ ^]^ report the formation of ionic polymers via a salt‐metathesis‐based release method using salicylate‐based ionic liquids.

**Figure 4:**
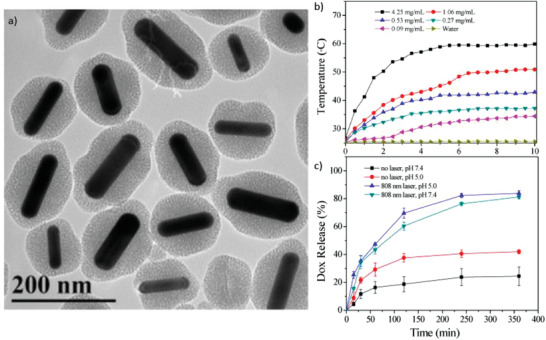
a) TEM images of magnetic core–IL shell microgels. b) Near‐IR‐induced heat generation of magnetic core–IL shell microgels. c) Doxorubicin release profiles from microgels with or without near‐IR laser irradiation at different pHs. Reproduced with permission.^[^ ^87^ ^]^ Copyright 2017, American Chemical Society.

ILs have also been conjugated to or combined with polymers to create delivery systems. Noshadi et al.^[^ ^84^ ^]^ report the use of choline based ILs to create conductive polymer‐gels with parameters, such as mechanical properties, porosity, and conductivity, that can be tuned by selection of the polymers and the ratio of polymer:IL. Sastry and coworkers^[^ ^76^ ^]^ prepared piperidinium‐based hydrogels with agar and chitosan, and they also demonstrated the tunability of the hydrogel, including the ability to modify drug release profiles. A choline chloride/xylitol DES was combined with a methacrylate polymer to encapsulate 5‐fluorouracil, which showed 77% encapsulation, and promising in vitro release, biodegradability, and toxicity against HeLa cells.^[^ ^85^ ^]^ Cellulose and chitosan were combined with imidazolium acetate to form an in situ film in mucin for the delivery of vitamin B12.^[^ ^86^ ^]^ Imidazolium cations with magnetic counterions were incorporated with deoxyribonucleic acid (DNA) cross‐linkers and gold nanorods into a microgel, which showed controlled release of chemotherapeutic doxorubicin when stimulated by a near infrared laser. Transmission electron microscopy (TEM) images of the particles, their temperature dependence, and release profile are shown in **Figure** **4**.^[^ ^87^ ^]^ Simpler doxorubicin‐IL systems have since been developed. Researchers made simple doxorubicin‐IL aqueous solutions that allowed for synergistic effects on CaCo‐2 cytotoxicity.^[^ ^88^ ^]^ ILs for the treatment of cancer have also been designed for controlled release of active pharmaceutical ingredients. By tuning the structure of the IL, researchers were able to control release based on NO uptake into the IL‐drug system.^[^ ^89^ ^]^

More recently, ILs have been applied as transport enhancers in drug delivery applications,^[^ ^90^ ^]^ where their interactions with the barrier tissues have enabled the delivery of large molecules through otherwise formidable barriers. Banerjee and coworkers^[^ ^91^ ^]^ delivered insulin orally using a choline geranate based ionic liquid, which maintains the secondary structure and activity of the insulin. Choline geranate has also been used to transport insulin transdermally, with the ion ratio affecting the delivery efficacy due to the mechanism of lipid extraction within the stratum corneum favoring a greater amount of “free” anion.^[^ ^92^ ^]^ This work was later expanded upon using biodegradable choline geranate‐containing patches that allowed for sustained systemic delivery of insulin through rat buccal tissue for safe non‐invasive, dose‐controlled delivery.^[^ ^93^ ^]^ Monti et al.^[^ ^94^ ^]^ utilized dilute (≈1% w/w) cyclic onium ILs to deliver calcium channel blocker diltiazem, demonstrating low cytotoxicity and enhanced delivery over the control. Wu et al.^[^ ^96^ ^]^ used a choline malic acid IL to deliver a 4000 Dalton dextran, both in vitro and in vivo in rats, showing a twofold enhancement in delivery to the dermis over the control and minimal cytotoxicity to human keratinocytes. Similar choline carboxylic acid ILs have been used to deliver small interfering ribonucleic acid topically for the treatment of psoriasis,^[^ ^96^ ^]^ antibodies orally,^[^ ^97^ ^]^ and peptides for vaccination.^[^ ^98^ ^]^ As ILs gain popularity as macromolecular transport vehicles, more comprehensive studies need to be performed to understand IL‐payload interaction and transport characteristics. A comprehensive study of two model drugs, one hydrophilic and one hydrophobic, was undertaken with 16 quaternary ammonium carboxylic acid ILs, and 2D nuclear magnetic resonance (NMR) was utilized to infer an inverse correlation between ionic components and transport efficacy.^[^ ^99^ ^]^ A compilation of the therapeutics used and the potential use cases of technologies discussed in this section can be found in Table S4, Supporting Information. The use of ionic liquids in a drug delivery context—either to improve existing vehicles or used independently—is in its early phase, and the potential impacts of this are high given the exceptional initial results for oral, transdermal, and buccal delivery. Many of the papers covered here, provide in vitro and in vivo efficacy of IL‐drug systems. However, understanding the biophysics of IL‐tissue interactions should allow the field to advance more quickly, to allow for the less reliance on slower throughput screening methods.

### 2.4 Nanocarriers

The use of ionic liquids within biomedical nanocarriers was initiated by their use for synthesis and templating of silica nanoparticles for antibacterial,^[^ ^100^ ^]^ gene transfection,^[^ ^101^ ^]^ and drug release applications.^[^ ^102^ ^]^ They were then later incorporated into nanocarriers as emulsifiers,^[^ ^34^, ^103^ ^]^ nanocomposite systems,^[^ ^104^ ^]^ and later in nanoparticle synthesis using materials other than silica.^[^ ^105^, ^106^, ^107^ ^]^ ILs have also recently been used in the synthesis of nanocarriers^[^ ^108^ ^]^ to improve many of their characteristics, including biocompatibility, stability, and drug loading. IL‐based nanocomposites have been used to increase drug solubility and efficacy of the anticancer drug rutin. Rutin has a strong promise as a treatment for renal cell carcinoma but is limited by its poor water solubility and pharmacokinetics. The researchers were able to encapsulate choline amino acid‐based ILs (phenylalanine and glycine) in poly(lactic‐*co*‐glycolic acid) nanoparticles and achieve similar toxicity of free rutin with a tenfold increase in drug concentration in water.^[^ ^109^ ^]^

An important factor that allows for increased drug solvation and a unique ability of ILs are their ability to self‐assemble into nanostructures in aqueous solution. This phenomenon was computationally explored using molecular dynamics by the Benedetto group. The found that at lower IL‐in‐water concentrations, less than 50 wt%, salt‐rich nanometer‐size domains form in water. However, at higher concentrations, above 50 wt%, the roles are flipped and water nanodomains are found in a sea of IL.^[^ ^110^ ^]^ Other groups have shown that these nano‐ and sometimes micro‐structuring effects can impact cytotoxicity of IL containing aqueous mixtures. They found that IL mixtures did not strictly follow a concentration dependent cytotoxic affect and that the micro‐structuring, as a result of combining multiple ILs, caused antagonistic behavior from the solutions.^[^ ^111^ ^]^ This unique nano‐ and micro‐structuring property has been utilized to solvate a number of particularly water‐insoluble drugs. Choline geranate was used to increase solubility and improve delivery of sorafenib, a liver and kidney cancer drug, to these tissues.^[^ ^112^ ^]^ More recently a similar formulation was used to improve sorafenib anticancer efficacy by fivefold. The increase in efficacy is caused by inhibiting exocytosis of the drug rather than increasing cellular uptake suggesting that the sorafenib‐choline‐geranic acid formulation may be a great formulation for tumor growth inhibition.^[^ ^113^ ^]^ Zainal‐Abidin et al.^[^ ^114^ ^]^ report the use of a choline chloride:malonic acid DES in improving the biocompatibility of tamoxifen‐loaded graphene, showing decreased cytotoxicity compared to bare graphene as measured by cell cycle progression, cell viability, and generation of reactive oxygen species. Sundrarajan et al.^[^ ^115^ ^]^ used an ammonium bistriflimide IL as a capping agent for silver/manganese oxide/bentonite nanocomposites. The use of the IL improved cytotoxicity over the bare nanocomposites when applied to a human embryonic kidney cell line (HEK293).

Safdar et al.^[^ ^116^ ^]^ also modified chitosan with ammonium‐based ILs, creating particles with much greater stability than chitosan alone. Azobenzene ILs were used as photosensitive molecular jacks that released stored cargo under UV radiation by molecular reconfiguration.^[^ ^117^ ^]^ An imidazolium IL was loaded into a zeolite‐based nano‐framework to enhance their hyperthermal, and thus tumor killing, properties.^[^ ^118^ ^]^ Also enhancing their thermal properties, Chen et al.^[^ ^119^ ^]^ incorporated a microwave‐sensitive IL into zirconia mitochondria‐targeted nanoparticles. A range of imidazolium ILs were also utilized to synthesize fluorescent carbon dots (CD), with the quantum yield depending on the length of the side chain on the imidazolium head group and the nucleophilic character of the anion.^[^ ^120^ ^]^ The resultant IL‐CDs showed increased transport of loaded curcumin into HeLa cells and greater cell death. Curcumin was also delivered in IL modified pH sensitive silica‐graphene oxide nanoparticles.^[^ ^121^ ^]^ Recently, Hamadani et al. developed protein avoidant choline carboxylic acid ILs to coat polymeric particles. The ionic liquid coating resisted serum absorption and redirected biodistribution of the injected particles to the lung via spontaneous hitchhiking onto red blood cells in situ.^[^ ^122^ ^]^

A quaternary ammonium cation was mixed with mefenamic acid, L‐lactide and a tin catalyst, resulting in the formation of cationic polylactide nanoparticles (NPs) which entrapped the mefenamic acid molecules.^[^ ^123^, ^124^ ^]^ Jahed et al.^[^ ^125^ ^]^ also formed cationic nanocomposite structures using a benzotriazolium IL monomer that showed pH dependent drug release. Similar pH responsive nanocarriers were synthesized by polymerizing an imidazolium IL with 2‐(dimethylamino)ethyl methacrylate and p‐hydroxyazobenzene (for light‐responsive characteristics) for controlled release of doxorubicin.^[^ ^126^ ^]^ Ammonium methacrylate polymers were complexed with either salicylate or sulfacetamide anions and a range of non‐ionic pharmaceuticals were encapsulated to create a matrix which was capable of releasing both ionic and non‐ionic molecules simultaneously.^[^ ^127^ ^]^ The best performing matrix showed a good release profile, low cytotoxicity to human cell lines, and antibacterial effects against *Escherichia coli*. A vinyl imidazolium bromide IL was used as a monomer to create polymer‐based brush nanomotors that are fueled by enmeshed smaller platinum NPs in a hydrogen peroxide environment.^[^ ^128^ ^]^ Umapathi et al.^[^ ^129^ ^]^ investigated the interactions between a thermoresponsive polymer and choline‐based ionic liquids, evidencing the impact of the concentration and orientation of the IL components on the phase behavior of the polymer.

**Figure 5:**
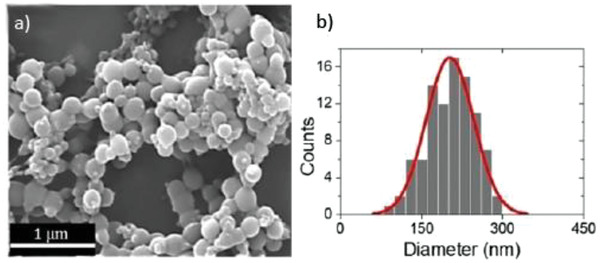
a) SEM image of BSA nanoparticles prepared by in IL. b) Particle size distribution obtained from the SEM image. Reproduced with permission.^[^ ^131^ ^]^ Copyright 2018, Wiley‐VCH.

A mixture of imidazolium tetrafluoroborate IL and choline chloride/ethylene glycol DES was used to facilitate the facile synthesis of doped single walled carbon nanotubes by dissolving and evenly distributing the starting materials.^[^ ^130^ ^]^ A water/imidazolium tetrafluoroborate solution was used to synthesize bovine serum albumin NPs with well controlled size, as seen in **Figure** **5**.^[^ ^131^ ^]^ This occurs because the albumin undergoes conformational changes at the IL/water interface, spontaneously forming particles of different sizes depending on the length of the side chain on the cation. Cellante et al.^[^ ^132^ ^]^ used a pyrrolidinium hydrogen sulfate IL as a solvent and a catalyst to link a peptide to nanocellulose using a Fisher esterification. Qi et al.^[^ ^133^ ^]^ prepared starch nanoparticles using a methylimidazolium acetate IL‐in‐oil emulsion. The IL in this case was selected on the basis of the high solubility of octenyl succinic anhydride maize starch, which enabled the synthesis of monodisperse particles with a narrow size distribution. ILs can also be utilized to easily synthesize externally controllable nanocarriers as such as the microwave controlled particles mentioned earlier^[^ ^118^, ^120^ ^]^ or magnetic nanocomposites comprised of Fe_3_O_4_ cores and Chitosan‐methoxypolyethylene glycol polymerized with a 2‐*N*‐benzylidene IL. The magnetic particles were used to selectively deliver a combination of chemotherapeutics, doxorubicin, and methotrexate.^[^ ^134^ ^]^ Magnetic nano‐emulsions of [ProC_10_][FeCl_3_Br] in water and showed promising in vitro release of hydrophobic model drugs.^[^ ^135^ ^]^ More detail about the ionic liquids used and their applications covered in this section can be found in Table S5, Supporting Information.

IL charge and amphiphilicity characteristics have aided in the synthesis of a variety of polymer and protein nanoparticles, making potential drug carriers easier and safer to produce than the conventional methods.^[^ ^115^, ^116^, ^117^, ^118^, ^119^, ^120^, ^121^, ^125^, ^128^, ^130^, ^131^, ^132^, ^133^ ^]^ In all, incorporating ILs into the synthesis or modification of nanocarriers is a promising emerging application for this solvent class. Careful selection of the IL components can impact the success of the synthesis procedure and the biocompatibility of the final material, and because the ILs retain their properties once placed onto the surfaces of nanoparticles, the tunability of ILs can be exploited to control the relationships between nanoparticles and their environments.

### 2.5 Protein Stabilization

Research on IL‐protein interactions began as an assessment of prevention of aggregation,^[^ ^136^, ^137^ ^]^ reversal of aggregation,^[^ ^138^ ^]^ and refolding^[^ ^139^ ^]^ with significant focus on long term stability.^[^ ^140^ ^]^ Investigations into the interactions involved in these systems^[^ ^141^, ^142^, ^143^ ^]^ became more relevant in order to determine the best ways to design ILs for stabilization of native proteins.^[^ ^144^ ^]^ Recently, ionic liquids have shown to interact with proteins,^[^ ^145^, ^146^, ^147^ ^]^ including enzymes,^[^ ^148^, ^149^, ^150^, ^151^ ^]^ with ammonium‐based ionic liquids in particular offering low toxicity and thermal and conformational stability.^[^ ^152^, ^153^, ^154^ ^]^ Although the interactions between proteins and other biological materials and ionic liquids are not yet perfectly understood, great progress has been made in this arena by uniting experimental and theoretical approaches.^[^ ^155^, ^156^, ^157^, ^158^, ^159^ ^]^ This subsection will describe the current state of knowledge of protein–ionic liquid interactions and how that knowledge has been usefully employed in applications.

The amphiphilicity of biocompatible ILs allows for unique properties when formulated with hydrophilic molecules like proteins. The use of ILs can elicit longer protein shelf lives and overcome some of the formulation hurdles faced by the traditional aqueous buffered solutions. Of particular interest is the stabilization of insulin amyloids which are important protein aggregates that have strong implications in therapeutic insulin treatment. Finding ways to prevent fibril formation and facilitate refolding of amyloids into monomers is extremely important to the field of medicine. Ishikawa et al.^[^ ^145^ ^]^ created aqueous formulations with 20% mol/mol of 1‐butyl‐3‐methylimidazolium hexafluorophosphate‐based ILs ([Bmim][SCN], [Bmim][NO_3_], and [Bmim][Cl]) that allowed for cryopreservation at −196 °C. This enabled extended stable storage of insulin amyloids and 80% recovery of insulin secondary structure after IL removal. Heyert et al.^[^ ^146^ ^]^ showed that ILs can enable special protein characteristics that cannot be attained in other traditional molecular solvents. The IL 1‐butyl‐1‐methylpyrrolidinium bis(trifluoromethysulfonyl)imide ([C_4_mpy][Tf_2_N]) was used to restrict the microstates of various Xaa‐Pro (any amino acid followed by a proline) dipeptides, which allowed for energetically unfavorable conformations to form that cannot be observed in water or octanol. Of particular interest was the Trp‐Pro dipeptide that was stabilized in the cis transformation in the IL.^[^ ^147^ ^]^ Testing various proteins has given insight into how the size and type of protein can affect IL interactions. Dilational rheology performed at the oil–water interface of [C_12_mim][Br] with BSA showed that increasing IL concentration drastically affected the BSA secondary structure. This suggests that BSA unfolds to allow for co‐absorption of the amphiphilic IL on the interfacial surface.^[^ ^147^ ^]^ Recently, mixtures of polymers and ILs have made an impact on protein stability. BSA thermal stability has subsequently been improved by 16 °C using polyethylene glycol and choline chloride urea.^[^ ^160^ ^]^ And *β*‐lactoglobulin experiences structural changes when exposed to choline iodide, but those effects can be negated by the addition of choline dihydrogen phosphate.^[^ ^161^ ^]^

**Figure 6:**
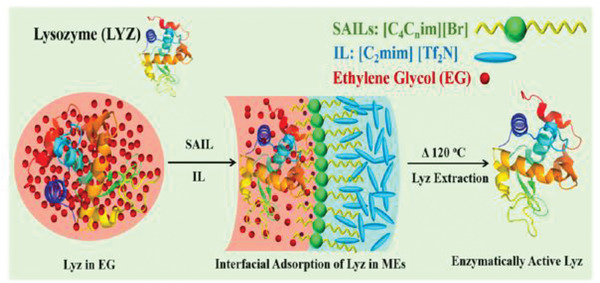
Schematic representation of lysozyme (LYZ) stabilization in surface active‐IL formulation. Reproduced with permission.^[^ ^148^ ^]^ Copyright 2019, American Chemical Society.

ILs have also shown great promise in enzyme activity retention. Lysozyme and Lipase are two enzymes that have been explored extensively. One paper outlines the use of microemulsions with ethylene glycol centers, dialkyl imidazolium‐based ([C_4_C_5_im][Br] or [C_4_C_9_im][Br]) surface‐active ionic liquids (SAILs) shells, and [C_2_mim][Tf_2_N] as a solvent to stabilize lysozymes. The formulations allowed for full enzyme activity even after heat treating at 120 °C, with the authors attributing the activity retention to the stabilization of Trp residues in the active site at the EG‐SAIL interface. A schematic of the lysozyme adsorption is shown in **Figure** **6**.^[^ ^148^ ^]^ Recent studies have been able to recreate these SAIL microemulsions without the need for ethylene glycol by utilizing dodecylbenzenesulfonate as an anion.^[^ ^162^ ^]^ Interactions near the active site play a key role in enzyme stability, as further supported by Zhao et al.^[^ ^149^ ^]^ who explored mutagenesis of lipase in the presence of ILs. They found that having positively charged amino acids near the catalytic site benefited enzyme stability in aqueous [Bmim][TfO] formulations by excluding [Bmim]^+^, whereas hydrophobic amino acids at the same location were deleterious to stabilization. IL solutions can do more than just stabilize enzymes—they have also shown potential in increasing their catalytic activity. Researchers increased enzymatic activity on day one of incubation by ≈25% using *N*‐ethyl‐*N*‐methylmorpholinium, triethylhexylammonium bromide, and other similar ILs at a concentration of 5% v/v. Additionally, they showed the IL formulations retained 90% enzymatic activity as compared to 70% in buffer for a four‐week room temperature incubation.^[^ ^150^ ^]^ A similar result was found by immobilizing peroxidase on an alginic acid derived DES material. The researchers saw a 5.5‐fold increase in enzyme activity with no deleterious effects.^[^ ^163^ ^]^ ILs have also been used to boost catalytic efficiency by functionalizing metal oxide nanoparticles. Iron oxide nanoparticles were functionalized with a chitosan‐linked 1‐(3‐aminopropyl)imidazolium coating for protein immobilization. They showed that these nanocomposites can increase the activity of porcine lipase by up to 6.68 times. Additionally, the nanocomposites allowed for higher retention of lipase activity after repeated cycles when compared to the control (90% activity in the IL‐modified composites compared to 70% in the unmodified case).^[^ ^151^ ^]^ Collagen thermal stability in an in vivo model was increased from 61 to 87 °C using cerium oxide‐choline serinate functionalized particles. These particles have potential use as a collagen crosslinking agent in vivo and for tissue engineering applications.^[^ ^164^ ^]^

Ammonium ILs’ nitrogen core positions them as unique compounds to solve stabilization issues for both enzymes as well as other therapeutic proteins. The formation of a protein‐IL gel was shown using 2‐hydroxyethyl ammonium formate and *ε*‐poly‐l‐lysine. Rheology showed solutions with 3.5 mg mL^−1^ of IL and 15% w/v polypeptide in water formed thermo‐reversible gels that allowed 90% retention of the secondary structure.^[^ ^152^ ^]^ Reasonably, one might think that incorporating amino acids into the IL would have favorable interactions with proteins. Guncheva et al.^[^ ^153^ ^]^ explored anions made from aspartic acid, glutamic acid, lysine, and arginine to create amino acid‐based ILs. They showed a decrease in *α*‐helical structure and an increase in *β* structures in insulin. [Cho][Glu] and [Cho]_2_[Asp] at concentrations of 10 mm in buffer showed the best results for insulin structural stability.^[^ ^153^ ^]^ There have also been studies to gain better insight into how exactly these ammonium ILs are interacting with proteins on an amino acid level. A [Choline Chloride][Glycerol] DES at a concentration of 10% v/v was used to test stability of versatile peroxidase in an aqueous IL solution. Using extrinsic fluorescence, the authors showed an increase in solvent‐exposed hydrophobic patches and *α*‐helical content. In the case of versatile peroxidase, this decreased thermal degradation and improved activity.^[^ ^154^ ^]^ The juxtaposition of the two previously mentioned papers highlights the importance of optimizing IL chemistry for the prevention of unwanted protein modifications and a better understanding of the microscopic protein‐IL interactions. A multitude of techniques need to be utilized for a more holistic understanding of protein stability. Bui‐Lee et al.^[^ ^165^ ^]^ used circular dichroism, fluorescence, UV/VIS and NMR spectroscopy, and small‐angle X‐ray scattering to provide a proper assessment green fluorescent protein stability in a variety of ILs.

Although the interactions between proteins and ionic liquids are not yet perfectly understood, great progress has been made in this area by combining experimental and theoretical approaches. The physicochemical properties, like hydrophobicity, hydrogen bonding ability, and ion tunability, and biopharmaceutical properties of ILs lead them to be suitable solvents for macromolecules, especially ones that have poor water solubility.^[^ ^156^ ^]^ Additionally, more focused literature reviews have been published that examine the use of IL mixtures that act synergistically in improving protein stability,^[^ ^166^ ^]^ the refolding of effects of ILs on proteins in vitro,^[^ ^167^ ^]^ and amyloidogenesis.^[^ ^168^ ^]^ This was shown through neutron scattering and molecular dynamics simulations with phospholipids, which suggests that electrostatics, hydrophobic, and hydrogen bonding are the major players in the IL‐phospholipid interactions.^[^ ^155^ ^]^ Arumugam et al.^[^ ^157^, ^158^ ^]^ have done extensive work in connecting theoretical and experimental results. BSA secondary structure was drastically affected by higher concentrations of [EPMpyr][Cl] in an aqueous formulation. Using steady state emission spectral studies, they elucidated that the BSA‐IL mixture had an increased number of hydrophobic and electrostatic interactions as compared to BSA in buffer. Molecular docking simulations supported this notion by revealing that the cation was able to penetrate the subdomains of BSA due to its hydrophobic nature, thus causing loss of secondary structure and increased electrostatic interactions.^[^ ^157^ ^]^ This theoretical work is further supported by experimental work performed with BSA and *N*‑2′,3′‑epoxypropyl‑*N*‑methyl‑2‑oxopyrrolidinium salicylate ([EPMpyr][Sal]).^[^ ^158^ ^]^ While molecular docking can give insights into the protein–IL interactions, molecular dynamics simulations are the gold standard for modeling behavior in solution. Molecular dynamics simulations showed that the 1‐hexyl 3‐methyl‐imidozolium bromide ([HMIMCI][Br]) bound to the glucose oxidase active site, especially at higher aqueous concentrations, which essentially deactivates the enzyme. Experimental studies corroborated the in silico findings when elevated concentrations of ILs inhibited the catalytic activity and denatured some enzyme tertiary structures. Despite this, these ILs might still be useful as a storage solvent because the overall structure retention, measured as RMSD, was better at higher IL concentrations.^[^ ^159^ ^]^ A summary of the relevant papers and their protein stabilizing performance can be found in Table S6, Supporting Information. As IL–protein interactions are better understood, new formulations can be developed to create shelf‐stable biologics that have enhanced treatment efficacy either through IL synergistic effects or to create localized, targeted biologic treatments. As researchers look to push the boundaries of protein–IL formulations by using proteins that are more complex, that is, larger and having varied secondary structures, elucidating the interactions between hydrophobic IL components and the non‐solvent‐exposed hydrophobic amino acid residues will be key. The challenge being to achieve the desired use cases, like therapeutic stabilization and drug delivery, hydrophobic ions are necessary but can cause misfolding and aggregation. While this could be solved by adding excipients to protect proteins from ILs, tunability should allow for the intelligent design of simple protein–IL formulations. Further assessment of biophysics of protein stabilization and IL–protein interactions in general will be extremely valuable in advancing the field.

### 2.6 Antimicrobial Agents

In the 2000s, ionic liquids showed promise as novel antimicrobial agents with research largely focused on development of ILs with imidazolium^[^ ^169^, ^170^, ^171^ ^]^ and quinolinium cations.^[^ ^172^ ^]^ Later, researchers began to more rigorously assess the surface interactions that made ILs such strong antimicrobial candidates.^[^ ^173^, ^174^ ^]^ Recently they have been both used alone^[^ ^175^, ^176^, ^177^, ^178^, ^179^, ^180^, ^181^, ^182^ ^]^ and incorporated into hybrid materials.^[^ ^183^, ^184^, ^185^, ^186^, ^187^, ^188^, ^189^, ^190^ ^]^ In particular, poly(ionic liquids) have recently shown excellent antibacterial properties^[^ ^191^, ^192^, ^193^, ^194^, ^195^, ^196^, ^197^, ^198^, ^199^ ^]^ and their polymeric character has been used to manufacture antibacterial membranes.^[^ ^200^, ^201^ ^]^ There are two main proposed mechanisms of action for ionic liquid bactericidal activity. Both mechanisms suggest electrostatic forces cause localization of the positively charged IL components to the negatively charged regions on the microorganism cell membrane/wall. Once the cation is in close proximately to the membrane, hydrophobic interactions between the cation side chains and cellular lipids cause the molecule to insert itself into the membrane. This theory is supported by the notion that suggests IL systems with weaker cation‐anion interactions create better antimicrobial agents because the cation more easily separates from the anion and inserts into the membrane.^[^ ^181^ ^]^ This mechanism was further investigated by Benedetto et al.^[^ ^202^, ^203^ ^]^ using neutron refraction experiments. The researchers showed that for both choline and imidazolium‐based ionic liquids, the cations irreversibly infiltrate the lipid layers of biomembranes and occupy around 2–10% of the bilayer volume. Benedetto and Ballone reviewed recent experimental and theoretical work to understand the effect of ILs on the properties of lipids found in biomembranes.^[^ ^204^ ^]^ Lipophilic anions have also been shown to insert themselves into the cell membrane.^[^ ^178^ ^]^ The actual cause of cell death is where the two theories split. The first, more widely accepted theory, is that the infiltration of IL into the cell membrane can cause the cell to release cytoplasmic content like potassium ions, DNA, and ribonucleic acid (RNA) leading to cell death.^[^ ^176^, ^178^, ^184^, ^187^, ^193^ ^]^ The other proposed mechanism suggests that insertion of the positively charged molecule causes neutralization of the negative zeta potential of the cell surface. This prevents proper cell growth and causes aggregation of cells, resulting in a much slower antimicrobial effect.^[^ ^177^, ^180^ ^]^ In any case, a recent study explored antibacterial efficacy based on ionic liquid size and can give useful guidance on antimicrobial IL design.^[^ ^205^ ^]^

Ionic liquids have been used in combination with other materials or as polymerized ILs (poly‐ILs) to create more potent antimicrobial formulations. Chitosan is one such material that has similar proposed antimicrobial mechanisms to ILs, however some researchers have postulated that chitosan can penetrate cells and cause DNA conformational changes, which prevents messenger RNA transcription.^[^ ^183^ ^]^ The combination of other materials or techniques, like cyclic main chain polymers made from isosorbide, hexanediol and terephthaloyl chloride, fluorescent diketopyrrolopyrrole, or gamma irradiation during corneal surgery, with ILs have created synergistic antibacterial and antifungal treatments.^[^ ^172^, ^190^, ^193^ ^]^ ILs have also been combined with standard gauze to create antimicrobial wound dressings. However, the mechanism of action of the ionic liquid used in this particular application is unique. By creating a benzimidazolium dipodal anion, the researchers created an Fe^3+^ chelating ionic liquid. The positive charge again allows for IL localization near the bacterial cell wall. The chelating “arms” allow for IL entrapment of Fe^3+^ near the cell surface, preventing cellular uptake, which leads to cell death caused by iron deficiency.^[^ ^206^ ^]^ Interestingly, the addition of metal ions can also be used with ILs to cause bacterial cell death. Ferrocene^[^ ^191^ ^]^ and zinc/copper^[^ ^201^ ^]^ containing ionic liquids and poly‐IL membranes allow for the generation of reactive oxygen species that cause oxidative damage to bacterial cell walls. Their higher localized positive charge and hydrophobic character has meant that poly‐ILs are more effective than their free cationic compound counterparts. Studies have found that poly‐IL efficacy is not anion dependent^[^ ^184^ ^]^ and that polymers with main‐chain ILs work better than side‐chain ILs.^[^ ^193^ ^]^ However, many of these hybrid IL materials and poly‐IL formulations have only been tested across two strains of bacteria.

A majority of the papers reviewed in this section utilize either *E. coli*,^[^ ^157^, ^160^, ^161^, ^170^, ^190^ ^]^ *Staphylococcus aureus*,^[^ ^184^, ^192^ ^]^ or both^[^ ^153^, ^154^, ^155^, ^156^, ^158^, ^162^, ^165^, ^166^, ^168^, ^169^, ^171^, ^173^, ^174^, ^176^, ^183^, ^184^ ^]^ to test IL/formulation bactericidal efficacy. The two bacterial strains are extremely clinically relevant, as these are both common bacterial infections in humans, and they *E. coli* and *S. aureus* represent two general categories of bacteria, Gram‐negative and Gram‐positive, respectively. However, the use of only two strains may misguide the conclusions of IL's antibacterial ability. Weyhing‐Zerrer et al.^[^ ^182^ ^]^ tested 12 strains of bacteria, 6 Gram‐negative and 6 Gram‐positive, with 28 different ILs to create a structure‐activity relationship. They found that bactericidal efficacy could not be generalized across strains of bacteria, even within the same Gram category, suggesting that there may be different mechanisms of action at play across different bacterial strains. There is also limited research on IL antifungal and anti‐mold performance. A few studies have reported antifungal efficacy with *Candida albicans* ^[^ ^156^, ^158^, ^166^, ^171^ ^]^ and anti‐mold studies were performed on *Asp. niger, Asp. oryzae*, and *Rhizopus*.^[^ ^188^ ^]^ An overview of the type of strains tested in these papers can be found in Table S7, Supporting Information.

With drastic strides being made in the use of ILs as antimicrobial agents, the coupling of these unique technologies with applications like active pharmaceutical ingredients, nanocarriers, and drug delivery agents could enable a new wave of combinatory therapeutics that serve as both disease treatments and antibiotics. However, current studies are narrow in scope when it comes to microbial species and researchers will need to explore other strains in addition to *S. aureus and E. coli* in order to truly evaluate IL antimicrobial efficacy.^[^ ^182^ ^]^ Additionally, efficacy experiments need to become more rigorous to ensure their ability to treat infections in vivo in animal models rather than simple in vitro microbial growth experiments.

### 2.7 Biosensing

The detection and quantification of biologically relevant molecules is extremely important to inform medical treatment and monitor patient health. The incorporation of ionic liquids into analytical biosensing platforms is amongst the most popular usage of ionic liquids in biomedical applications.^[^ ^208^ ^]^ Within electrochemical sensors, ILs, because of their inherent conductivity,^[^ ^209^ ^]^ are used as electrolytes to allow for sufficient electron flow between the analyte detection, either through bioreceptor interaction or reduction‐oxidation reactions in the analyte, and the electrochemical transducer. In the mid to late 2000s this often required carbon precious metals^[^ ^210^, ^211^, ^212^ ^]^ but has shifted in favor of carbon nanotubes^[^ ^213^, ^214^, ^215^ ^]^ and graphene^[^ ^216^, ^217^, ^218^ ^]^ for more cost effective sensors. To this end, almost all of the electrochemical sensors mentioned here are comprised of a carbon electrode coated in an IL, with bioreceptor macromolecules or nanoparticles immobilized on the surface. The analytes that these electrochemical sensors aim to detect range from biomarkers to therapeutics to omnipresent organic molecules.^[^ ^219^, ^220^, ^221^, ^222^, ^223^, ^224^, ^225^, ^226^, ^227^, ^228^, ^229^, ^230^, ^231^, ^232^, ^233^, ^234^, ^235^, ^236^ ^]^ As mentioned previously, imidazolium and cholinium‐based ionic liquids have been widely used in biomedicine, which holds true for biosensors. Choline dihydrogen phosphate and 1‐butyl‐3‐methylimidazolium tetrafluoroborate (BMIM[BF_4_]) coated electrodes with immobilized antibodies were used to detect cortisol and interleukin‐6 in human sweat. Detection of these two biomarkers in sweat gives insight into underlying health conditions and could allow for 24/7 health monitoring for patients.^[^ ^223^ ^]^ This work was further expanded to create a sensor that could detect cortisol in sweat, urine, and saliva.^[^ ^237^ ^]^ Other diagnostic biosensors have been used to detect small organic molecules that have been classically used to gauge a patient's health. One such sensor was created to detect cholesterol by electrodeposition of 1‐butyl‐3‐methylimidazolium chloride (BMIM[Cl]) on a carbon electrode with cholesterol oxidase immobilized on the surface. The researchers were able to achieve much lower limit of detection (LOD) and limit of quantification (LOQ) with faster readout times than classical quantification methods like high‐performance liquid chromatography (HPLC).^[^ ^234^ ^]^ However, small molecule biosensing is not limited to ubiquitous molecules and has found great use in quantification of drugs present in the serum. Thiopurines, common anti‐cancer and autoimmune disease treatments, can cause off‐target toxicity and other complications when the levels in the serum are too high. Thus, monitoring of serum levels of these drugs is extremely important during treatment. Shpigun et al.^[^ ^230^ ^]^ created a biosensor using 1‐butyl‐3‐methylimidazolium hexafluorophosphate (BMIM[PF_6_]) and ZnO nanoparticles to detect three common thiopurines, 6‐thioguanine, 6‐mercaptopurine, and azathioprine. The nanoparticles electrooxidized the drugs and the IL allowed for signal transduction. The benefit of IL‐based biosensors is that conscious IL design and bioreceptor selection can allow for detection of a vast number of biologically relevant molecules. Table S8, Supporting Information, summarizes analytes and detection limits of recent IL‐based sensing applications.

Another context in which ILs have been used is as chemical extractants in chemiluminescent and fluorescent sensors. Since most biologically relevant molecules are in aqueous solutions, like serum, many researchers have started designing hydrophobic ILs in order to separate molecules of interest from the bulk solution. Once the analyte is extracted it can be analyzed using various fluorescent and chemiluminescent techniques. Zeeb et al.^[^ ^238^ ^]^ utilized reactive ionic liquids to achieve sufficient extraction of duloxetine, an antidepressant that can cause suicidal thoughts when serum levels are too high, and meloxicam, an non‐steroidal anti‐inflammatory drug (NSAID) that has implications in joint diseases like rheumatoid arthritis, from serum. Plasma samples were spiked with NaPF_6_ and BMIM[BF_4_] before sonication which caused the formation of a BMIM[PF_6_] microemulsion in which the two drugs were dissolved. These hydrophobic phases could then be easily separated using centrifugation and analyzed using reverse‐phase high‐performance liquid chromatography (RP‐HPLC). Significantly low detection limits of 0.8 *μ*g mL^−1^ and 1 ng mL^−1^ for duloxetine and meloxicam, respectively were achieved.^[^ ^238^, ^239^ ^]^ Similarly, poly(IL) coatings of 1‐vinyl‐3‐octylimidazolium hexafluorophosphate have been used to surface modify silica‐coated iron oxide nanoparticles to make magnetic extraction devices. When vigorously mixed with serum, these magnetic poly(IL)‐coated nanoparticles allow for extraction of three antidiabetic drugs (empagliflozin, metformin, and canagliflozin). The nanoparticles can then be washed with acetonitrile and drug concentrations can be quantified with RP‐HPLC.^[^ ^240^ ^]^ Similar magnetic techniques have been used to separate heme proteins, cephalosporins, and NSAIDs from biological fluids.^[^ ^241^ ^]^ Electrogenerated chemiluminescent sensors have allowed for quantification of ethanol through the use of alcohol dehydrogenase oxidation. The sensor utilizes BMIM[Cl]‐titania‐Nafion coated glassy carbon electrode with inner layers of Ru(bpy)_3_^2+^ and outer layers of alcohol dehydrogenase. The Ru(bpy)_3_^2+^ is a regenerable lumiphore that allows for repeated biosensor use.^[^ ^242^ ^]^ As previously mentioned, detection of small molecules that can be leached from common items like polymers and textiles can be extremely important to confirm product safety. One such relevant molecule, p‐phenylenediamine (PPD), can cause DNA damage in human cells. Block copolymer micelles made of an amphiphilic poly‐IL and hydrophobic fluorescent 1‐pyrenecarboxaldehyde have been used to quantify PPD in aqueous solutions. A condensation reaction between PPD and 1‐pyrenecarboxaldehyde quenches the fluorescence in the micelles.^[^ ^246^ ^]^ Applications have been further advanced to create two way “on‐off” fluorescent sensing of biorelevant material. The sensors that are made from N‐doped graphene quantum dots autofluoresce in 1‐hexyl‐3‐methylimidazolium tryptophan. In the presence of Cu^2+^, the fluorescence is quenched and “turned off.” The sensor can subsequently be turned back “on” with levodopa, a precursor of dopamine that is used to treat Parkinson's. Both Cu^2+^ and levodopa can be quantified by fluorescence spectrophotometry.^[^ ^247^ ^]^ And in a more macroscopic application, 1‐ethyl‐3‐methylimidazolium bis(trifluoromethylsulfonyl)imide has been used to create an ultra‐sensitive, flexible pressure sensor skins.^[^ ^245^ ^]^ As ionic liquids improve biosensor technology, the ability to detect markers of disease and tightly control drug doses will allow doctors to more easily and successfully treat disease. The main challenge with moving this technology forward is the ability to easily deposit IL molecules on surfaces for use in analytical devices. Recent advances have used electrodeposition to immobilize ILs on various electrodes. However, this creates challenges when the detection is based on a biological recognition event, that is, enzyme reaction or protein binding, because the bioreceptor cannot be immobilized at the same time as the IL layer. Thus, finding a way to simultaneously deposit ILs and bioreceptors will allow for improved sensors and simple detection of new molecules. The field of biosensors aims to achieve “ultrasensitive” detection, that is, picomolar detection of biorelevant molecules, but is hindered by sensitivity and selectivity issues of bioreceptors. But the recent advances in IL‐mediated protein stabilization might allow for conformational control over bioreceptors, like enzymes, so that sensors can achieve lower detection limits.

### 2.8 Challenges and Future Outlook

#### 2.8.1 Biocompatibility

Work is ongoing to discern the toxicological impacts of ionic liquids,^[^ ^246^ ^]^ but much remains to be done to ensure their long‐term safety. Like any other chemical, ionic liquids face the challenge of limited applicability of in vitro systems to predict in vivo outcomes. This problem is further magnified by the limited scope of in vitro studies that have been utilized, for example the ubiquitous use of only *E. coli* and *S. aureus* for antibacterial assessment or the use of single cell lines to assess toxicity. Translation of studies in small animal models to humans also remains to be seen. Safety studies need be performed with ionic liquids as well as their final formulations to be administered. However, the risk of potential toxicity can be partially mitigated by utilizing ions that are natural metabolites or have been approved for use in food or pharmaceutical materials. For example, choline and many carboxylic acids are part of the normal human diet and have been utilized in clinically approved formulations, respectively. While this can provide a good starting point for researchers and drug developers, for ionic liquids to become relevant treatment components, regulatory agencies will want to see clinical data to justify the safety of ionic liquids and their components at the administration site. As techniques of assessing biocompatibility across all of biomedicine advance, with promised future organ and human on a chip assays, it should allow for more streamlined assessment of ionic liquid toxicity studies.

#### 2.8.2 Impact of Impurities

A focus of much of the earlier generation of ionic liquid was the role that even minor impurities play in the properties of the solvent, including the impact of the presence of water, halides, and other starting materials. Very little attention has been paid to the impact of trace impurities on the properties of the so‐called third generation bio‐compatible ionic liquids and their biological interactions. These impurities can significantly affect the characteristic material properties that were mentioned earlier and make ionic liquids a unique class of materials. One article found that a 10% increase in molar water concentration can cause a 30% decrease in viscosity.^[^ ^247^ ^]^ Thus, it is necessary that researchers properly characterize and quantify impurities in ionic liquid formulations. Water content can easily be quantified, on a mass basis, utilizing Karl Fischer coulometer titrators. While water can generally affect the properties of the ionic liquid in question, it shouldn't be harmful in an in vivo setting. However, this is not always the case for unreacted precursors or halides which need to be quantified with great scrutiny before administration in vivo. While there are a number of techniques that could be utilized, which may depend on the particular molecules one is attempting to detect, HPLC to determine precursor content and Volhard titration to quantify halide impurities are commonly used in other areas within ionic liquid research.^[^ ^248^ ^]^ The role of impurities can also be circumvented somewhat by modifying the synthetic procedures to exclude potential interferants. By using hydroxyl or bicarbonate ions, the byproducts generated are carbon dioxide and water, which are both more easily removed than halide contaminants, and more compatible in trace amounts. The risk of unwanted impurities can also be mitigated by carefully designed synthesis. Employing precursors that react to produce byproducts like water or gases like carbon dioxide can allow for clean and simple production of the ionic liquids without impurities or ones that can be removed with simple techniques like rotary evaporation.

#### 2.8.3 Developing a Microscopic Understanding to Enable Task‐Specific Design

One of the biggest attractions of ionic liquids also presents one of the biggest hurdles in their rational design—their inherent tunability. Without an understanding of the microscopic interactions both within the solvent and between the solvent and any biological material, screening of ionic liquids must be done by brute‐force and without knowledge of possible broader implications of altering the ionic components. Literature reports of formulation supramolecular analysis are generally lacking, however limited available literature on this topic shows that there can be very interesting, unexpected structures that form when ionic liquids are mixed with other solvents or drugs. Understanding the structural behavior of the formulation after injection, topical application, or oral administration is extremely important for determining their interaction with the local tissues. It can also give insight into the mechanisms of action for antimicrobial applications and protein stabilization. Some initial insights have been achieved with 2D NMR and dynamic light scattering, but more substantial experiments will be required to establish a clearer picture at the microscopic‐level interactions, especially within the human body. Recent reports, have shown the impact that ionic liquids can have on cell migration in vivo. The study suggests that ILs can cause an increase cell migration rate through a localized area by affecting cell membrane elasticity.^[^ ^249^ ^]^ This is the sort of mechanistic and microscopic research that can help provide fundamental knowledge to ionic liquid design. More studies similar to this one with a large library of ionic liquids would provide a great service to all biomedical ionic liquid research.

## Conflict of Interest

S.M. and E.E.L.T. are inventors on several patents covering ionic liquids (owned by either University of California, Santa Barbara or Harvard University). S.M. is a consultant/board member/shareholder of Cage Bio, Liquideon LLC, and i2O Therapeutics.

## Supporting information

Supporting Information
