# Supplementary material for: Recent Advances in Ionic Liquids in Biomedicine
Source: Adv Sci (Weinh). 2021 Jul 10;8(17):2004819. doi: 10.1002/advs.202004819 (PMC8425867; doi:10.1002/advs.202004819)
Supplement: Supplementary file 1 — Supporting Information [file ADVS-8-2004819-s001.pdf]

© 2021 Wiley-VCH GmbH

ADVANCED SCIENCE
Open Access

Supporting Information

for *Adv. Sci.*, DOI: 10.1002/advs.202004819

Recent Advances in Ionic Liquids in Biomedicine

Alexander M. Curreri, Samir Mitragotri, and Eden E. L. Tanner**

Supporting Information Table 1*. Breakdown of the use of ILs discussed in each section, Solubilization and API-ILs are broken down by therapeutic class. Drug Delivery, Nanocarriers, and protein stabilization are broken down by application. Antimicrobial applications are broken down based on the strains that were tested, and biosensors broken down by classes of analytes. The references are color coordinated based on the IL class that was used in the following manner:* Imidazolium, Choline/Quaternary Ammonium, Pyrrolidinium, Pyridinium, Dual API-IL, Amino Acid, Various/Other.

Solubilization	
Anti-inflammatory	36, 41
Antimicrobial	29, 30, 31, 32, 33, 34, 35, 41
Anti-cancer	30, 37, 41
Various/Other	28, 29, 38, 39, 40, 41, 42, 43

API-ILs	
Antimicrobial	44, 47, 48, 50, 51
Anti-inflammatory	46, 46, 49, 50, 52, 54, 56, 58, 59, 60, 61, 62, 63
Anti-cancer	55, 57
Anesthetic	46, 49, 59, 61
Various/Other	45, 53

Drug Delivery	
Skin Delivery	65, 66, 67, 73, 80, 92, 94, 95, 96, 98, 99
Sustained Release	76, 78, 82, 83
Controlled Release	68, 69, 70, 71, 72, 74, 75, 88, 89
Responsive Release	75, 81, 84, 87
Oral Delivery	90, 91, 97
Gene Delivery	64, 67, 99
Other	79, 85, 86, 93

Nanocarriers	
Synthesis	101, 102, 105, 106, 130, 131, 132, 133
Emulsions	34, 103, 107, 110, 111, 112, 113
Responsive/Controllable Nanocarriers	118, 119, 121, 125, 126
Nanocomposites/Surface Modification	100, 101, 102, 104, 105, 109, 114, 115, 116, 117, 120, 122, 123, 124, 127, 129, 134
Other	108, 128, 135

Protein Stabilization

Antimicrobial

Thermal Stabilization	136, 138, 148, 153, 160, 161, 166
Therapeutic Proteins	145, 152
Enzyme Catalysis	139, 140, 142, 147, 148, 149, 150, 151, 154, 159, 162, 163
Interaction Assessment	141, 143, 146, 155, 156, 157, 158, 164, 165
Various/Other	137, 144, 167, 168

Biosensors

Glucose	210, 211, 214, 216, 230
Biomarker	212, 213, 219, 222, 226, 227, 231, 232, 235, 237
Therapeutic	215, 218, 221, 222, 223, 224, 228, 234, 236, 238, 239, 240, 244
Internalized Chemicals	217, 220, 229, 233, 242
Various/Other	241, 243, 245

Gram-positive	176, 184, 189, 205
Gram-negative	169, 174, 177, 187, 195
Both	170, 171, 172, 173, 175, 178, 179, 180, 181, 182, 183, 185, 186, 188, 190, 191, 192, 193, 194, 196, 197, 198, 199, 200, 201, 206
Fungi	170, 172, 173, 175, 183, 188, 198, 207
Mold	188
Membrane Interaction Assessment	202, 203, 204, 205

Supporting Information Table 2. Solubilization of Drugs

Reference Number	***Drug Class***	***Drug***	***Ionic Liquid(s) Used***	***Water Solubility Improvement (fold increase)***	***Findings/Application***
28	Other	Nucleoside	1-Methoxyethyl-3-methylimidazolium trifluoroacetate, other Imidizolium, and Pyridinium-based ILs	>100	Data suggested that ILs could be great solvents for sparingly soluble drugs
29	Anti-parasite<br>Hormone	Albendazole<br>Danazol	1-butyl-3- methylimidazolium hexaflourophosphate	>10000<br>>30000	Showed that altering imidazolium-cation structure can drastically affect solubility results
30	Antiviral<br>Chemotherapeutic	Acyclovir<br>Methotrexate	1,3-Dimethylimidazolium dimethyl phosphate	Not specified	Drug solubility in aqueous solution can be increased with increasing IL concentrations
31	Antibiotic	Erythromycin	Imidazolium, phosphonium, quaternary ammonium, and pyrrolidinium ILs	Not specified	Assessed thermodynamic properties of drug solubilization
32	Antifungal	Amphotericin B<br>Itraconazole	1-ethyl-3-methyl-imidazolium acetate<br>[m-PEG350-NH3][C9COO]	>400,000<br>>5,000,000	Improved solubility of anti-fungal drugs
33	Antifungal	Nimesulide	1-ethyl-3 methylimidazolium tetrafluoroborate	Not specified	Understanding the IL-drug interactions and how they affect binding
34	Antiviral	Acyclovir	Acetate anion with quaternary ammonium and imidazolium cations	>20-fold	Anti-viral drug used for herpes infections
35	Antiviral	Acyclovir	Cholinium glycinate and other choline amino acid ILs	>550-fold	
36	Anti-inflammatory	Ibuprofen	[C4C1im][N(CN)2]	120-fold	
37	Chemotherapeutic	Paclitaxel	Cholinium glycinate and other choline amino acid ILs	>5000-fold	Similar cancer cytotoxicity to current formulation without the need for CrEL (used for solubilization but has negative side effects)
38	Antidepressant	Nortriptyline Hydrochloride	1-decyl-3-methylimidazolium chloride		Thermodynamic and Supramolecular Characterization
39	Anesthetic	Lidocaine<br>Procaine	1-dodecyl-2,3-dimethylimidazolium chloride	N/A	Investigation of interactions involved in the IL system
40	Natural	Curcumin	cholinium oleate	>700	Enhanced water solubility with low (1 mg/mL) concentration of IL

Reference Number	*Drug Class*	*Drug*	*Ionic Liquid(s) Used*	*Water Solubility Improvement (fold increase)*	*Findings/Application*
41	Anti-inflammatory<br>Antiviral<br>Chemotherapeutic<br>Muscle Relaxant	Celecoxib<br>Acyclovir<br>Methotrexate<br>Dantrolene sodium	1,3-dimethylimidazolium dimethyl phosphate	>600-fold<br>35-fold<br>>300-fold<br>60-fold	Improvement of a variety of small molecule drugs in IL aqueous solution
42	Anticonvulsant	Lamotrigine	1-octyl-3-methylimidazolium bromide	N/A	Analysis of solvation and thermodynamic functions of the IL-water-drug system
43	Cardiovascular Drugs	LASSBio-294	1-ethyl-3-methylimidazolium methyl phosphonate and other imidazolium ILs, Ethylammonium acetate	N/A	Computational study of IL-interactions with a cardiovascular drug

Supporting Information Table 3. API-ILs

Reference Number	*Drug Class*	*Active Ingredient*	*Counter Ion*	*Water Solubility Improvement (fold increase)*	*Findings/Application*
44	Anesthetic	Lidocaine	Docusate	decreased solubility by >15	Showed API-IL formulation increased efficacy of drug *in vitro* compared standard formulation
45	Various	Various small molecule drugs	Various counter ions	N/A	A position paper that suggests API-ILs have the potential for enhanced delivery of small molecules
46	Anti-inflammatory	Aspirin (as salicylate or acetylsalicylate)	various counter ions	N/A	Doubly effective ILs with antibacterial, analgesic, local anesthetic, and antiarrhythmic cations
47	Antibiotic	Ampicillin	Choline	~10	Enhanced water solubility at physiological temperature
48	Antibiotic	β-Lactam	1-hexadecyl-3-methylimidazolium and 1-hexadecyl-2,3-dimethylimidazolium	Poorer water solubility	Allowed for decreased antibacterial IC50 concentrations of β-Lactam *in vitro*

Reference Number	*Drug Class*	*Active Ingredient*	*Counter Ion*	*Water Solubility Improvement (fold increase)*	*Findings/Application*
49	Anesthetic<br>Anti-inflammatory	Lidocaine<br>Ibuprofen	N/A	N/A	Faster release of both therapetuic molecules
50	Anti-inflammatory<br>Antimicrobial	Ibuprofen<br>Benzalkonium	N/A	N/A	Characterization of a API-IL with two active components
51	Antimicrobial	Benzethonium chloride	L-proline	N/A	Analysis of IL characteristics and biomolecule interactions
52	Anti-inflammatory	Salicylic Acid	1-hexyl-3-methylimidazolium	N/A	Thermophysical property study
53	Beta blocker (heart disease)	Carvedilol	Citric Acid, Tartaric Acid, Saccharin	2	Potential improved treatment of high blood pressure/heart failure
54	Anti-inflammatory	Mefenamic acid	Choline, ammonium ions	N/A	Development of a potential oral solid or gel NSAID
55	Chemotherapeutic	Methotrexate	quaternary ammonium, imidazolium, phosphonium, and amino acid derived cations	>5000	New formulation for common chemotherapeutic
56	Anti-inflammatory	Diclofenac	Imidazolium	42	Analgesic and Anti-inflammatory
57	Anti-cancer	Alendronic Acid	C2OHMIM, and other quaternary ammonium cations and superbases	>2000	Improved anti-cancer efficacy (specifically osteosarcoma)
58	Anti-inflammatory	Salicylic Acid	L-Proline ethyl ester and other Amino Acid Esters	>500	Used as model drug
59	Anti-inflammatory	Salicylic Acid	3-heptyl-1-vinylimidazolium	N/A	Microneedle IL-drug structures for topical acne treatment
60	Anesthetic<br>Anti-inflammatory	Lidocaine<br>Ibuprofen	N/A	N/A	Two incorporated drugs for anesthetic and anti-inflammatory topical treatments
61	Anesthetic<br>Anti-inflammatory	Lidocaine<br>Ibuprofen	N/A	N/A	Sustained release of lidocaine and ibuprofen over 10 days
62	Anti-inflammatory	Ibuprofen	1-butyl-3-methylimidazolium	>1000	Topical delivery of ibuprofen
63	Anti-inflammatory<br>Anti-inflammatory<br>Anti-inflammatory<br>Anti-inflammatory	Tolfenamic Acid<br>Meclofenamic Acid<br>Diclofenac<br>Ibuprofen	Quaternary ammonium ions	at least 50 in all cases	Demonstration of API-IL forming capabilities of weakly acidic compounds

Supporting Information Table 4. Drug Delivery

Reference Number	*Delivery Application*	*Drug/Payload of Interest*	*Ionic Liquid(s) Used*	*Findings/Application*
64	Gene Delivery	plasmid DNA	3-butyl-1-vinylimidazolium L-proline	Enhanced transfection of plasmid into cell with minimal decrease in cell viability for gene delivery
65	Skin	N/A	1-butyl-3- methylimidazolium hexafluorophosphate and 1-hexyl-3-methylimidazolium chloride	Topical delivery of fluorescently labeled model drug
66	Skin	Cefadroxil	choline geranate	Biofilm removal and transdermal antibiotic delivery
67	Skin/ Gene Delivery	RNAi	Benzyl dimethyl octyl ammonium	Transdermal delivery of RNAi for treatment of skin diseases
68	Controlled Release	Ibuprofen	1-methyl-3-butylimidazolium ibuprofenate	API-IL ionogel for controlled release of anti-inflammatory
69	Controlled Release	N/A	tetrabutylphosphonium styrenesulfonate and tributyl-hexyl phosphonium 3-sulfopropylacrylate	Thermo-responsive IL gel for potential controlled release
70	Controlled Release	N/A	Choline Chloride. Malonic Acid	Assessment of IL-water interactions and micelle formation
71	Controlled Release	N/A	[Cholesterol Glycine][AOT]	Assessment of IL-water interactions and micelle formation, with some ongoing work as drug carriers
72	Controlled Release	N/A	1-ethyl-3- methylimidazolium acetate	Formed stable microemulsions that showed anticancer activity
73	Skin	Acyclovir Methotrexate	triethylammonium acetate and diethylammonium acetate	Microemulsion drug carriers for sparingly soluble drugs, with potential transdermal applications
74	Controlled Release	Tetracaine	1-hexyl-3-methylimidazolium dodecylsulfate	Characterization study for drug-IL interactions to elucidate supramolecular structures
75	Responsive Release	Vitamin E	1-Hexadecyl-3-Methylimidazolium chloride	Bile Salt triggered dissociation of IL microemulsions for potential targeted release of vitamin E (and other drugs)
76	Controlled Release	Dexamethasone	4-dodecyl-4- methylmorpholinium chloride, 1-dodecyl-1-methylpyrrolidinium chloride	Potential delayed release of poorly water-soluble corticosteroid
77	Responsive Release	Camptothecin	choline chloride/citric acid DES	pH dependent delayed release of camptothecin
78	Sustained Release	Piroxicam	1-hexyl-3-methylimidazolium chloride and 1-butyl-3-methylimidazolium hexafluorophosphate	For sustained analgesic release (over 50 hours)
79	Various/Other	Amphotericin B	dicholinium bis(trifluoromethanesulfonyl)imide	A potential IV antiviral treatment (normal formulation causes hemolysis)

Reference Number	*Delivery Application*	*Drug/Payload of Interest*	*Ionic Liquid(s) Used*	*Findings/Application*
80	Skin	Dencichine	1-hydroxyethyl-3-methylimidazolium chloride and 1-butyl-3-methylimidazolium dodecanesulfate	Topical delivery of dencichine (potential hemostatic abilities)
81	Responsive Release	Aspirin	1- allyl-3-methylimidazolium chloride	pH-sensitive controlled release system of model drug aspirin
82	Sustained Release	Ranitidine hydrochloride	1-Ethyl-3-methylimidazolium acetate	Sustained release over 10h from gastric-floating gels
83	Sustained Release	Salicylic Acid	Poly[trimethylammonium (meth)acrylate] salicylate	Sustained release from API-IL polymer
84	Responsive Release	N/A	choline based poly-IL	Electrically conductive biodegradable hydrogels for drug delivery or tissue engineering
85	Sustained Release	Acetylsalicylate	1-dodecyl-1-methylpiperidinium acetylsalicylate	IL micelles were loaded into different viscoelastic gels for delayed release
85	Other	5-fluorouracil	choline chloride xylitol	Enhanced anti-cancer of 5-fluorouracil
86	Other	Cyanocobalamin	1-ethyl-3-methylimidazolium acetate	Sublingual delivery for treatments of vitamin B12 deficiency
87	Responsive Release	Doxorubicin	3-n-butyl-1-vinylimidazolium trichloromonobromoferrate	Magnetic, Near-infrared triggered nanocomposites allow for externally controllable release of chemotherapeutics
88	Controlled Release	Doxorubicin Mitoxantrone	Various 1-alkyl-3-methylimidazolium ILs	Analysis of drug solubility in various ILs mixtures
89	Controlled Release	NO	Various choline, imidazolium, and taurine based ILs	Controlled release of NO for inhibition of tumor growth
90	Oral	Selurampanel	Alkyl, Benzyl, Hydroxyl, Dication and Trication molecules with selurampanel anions	Oral delivery of sparingly water-soluble drug with controllable bioavailability based on cation used
91	Oral	Insulin	Cholinium geranate	Oral insulin delivery system
92	Skin	Insulin	Cholinium geranate	Transdermal insulin delivery system
93	Other	Insulin	Cholinium geranate	Buccal insulin delivery
94	Skin	Diltiazem	various 1,4-diazabicyclo[2.2.2]octane-based ILs	Potential transdermal delivery system for high blood pressure
95	Skin	FITC-Dextran	cholinium malate	Drug model showed potential for transdermal delivery of hydrophilic macromolecules
96	Skin/Gene Delivery	siRNA	Cholinium geranate and cholinium hydrocinnamate	Topical delivery of NFKBIZ siRNA for treatment of psoriasis
97	Oral	mAb	Cholinium glycolate	Potential for oral delivery of monoclonal antibodies

Reference Number	*Delivery Application*	*Drug/Payload of Interest*	*Ionic Liquid(s) Used*	*Findings/Application*
98	Skin	Antigen peptide	Cholinium -fatty carboxylic acid	Potential transdermal vaccine
99	Skin	Ruxolitinib<br>Acarbose	A variety of choline and quaternary ammonium with carboxylic acid ILs	Demonstrated that interactions between ions can give indication of transdermal potency

Supporting Information Table 5. Nanocarriers

Reference Number	*Nanocarrier Application*	*Therapeutic Payload*	*Nanomaterial*	*Ionic Liquid(s) Used*	*Findings/Application*
100	Nanocomposites	IL	Silica	1-alkyl-3- methylimidazolium tetrafluoroborate	Controlled release of IL from surface of silica nanoparticles for antibacterial purposes
101	Synthesis and Surface Modification	Plasmid DNA	Mesoporous silica	1-alkyl-3-methylimidazolium based IL	IL was used for synthesis and templating of the nanoparticles
102	Synthesis and Surface Modification	Ibuprofen	Silica	1-butyl-3-methylimidazolium tetrafluoroborate	Ionic liquid used to synthesize particles for extended release of ibuprofen for weeklong release
34	Emulsion	Acyclovir	IL-in-oil emulsion	dimethylimidazolium dimethylphosphate	Increased solubility and delivery of model drug
103	Emulsion	Dopamine hydrochloride<br>Acetylcholine chloride	IL micelles	1-tetradecyl-3-methylimidazolium bromide	Assessment of IL-drug interactions revealed importance of π-π interactions
104	Nanocomposites	Doxorubicin	poly(ionic liquid-co-N-isopropylacrylamide)	1-ethylvinyl pyridinium deoxycholate	pH and temperature responsive nanocarriers for potential targeted delivery of chemotherapeutics
105	Synthesis and Surface Modification	N/A	europium(III)-doped gadolinium(III) fluoride	1-butyl, 2- methylimidazolium tetrafluoroborate	IL used for synthesis and surface modification of quasi spherical biosafe nanoparticles
106	Synthesis	Curcumin	poly(2-hydroxyethyl methacrylate)	choline formate	Enhanced cytotoxicity of curcumin in cancer cell line, IL used for synthesis
107	Emulsion	Mitoxantrone hydrochloride	Starch	1-butyl-3-methylimidazolium hexafluorophosphate	Starch nanoparticles synthesized within IL-in-water microemulsion
108	Various	Various	Various	Various	Review focuses solely on ionic liquid incorporation into nanocarriers

Reference Number	*Nanocarrier Application*	*Therapeutic Payload*	*Nanomaterial*	*Ionic Liquid(s) Used*	*Findings/Application*
109	Nanocomposites	Rutin	PLGA	Cholinium Phenylalaninate and Cholinium Glycinate	IL-NPs increases the water solubility of the anti-renal cell carcinoma drug
110	Emulsion	N/A	IL-in-water, water-in-IL	Phosphonium and choline-based	Important molecular dynamics study to assess the types of structures that are formed in IL-aqueous solutions
111	Emulsion	N/A	N/A	Various 1-alkyl-3-methylimidazolium ILs	Found that IL cytotoxicity was did not strictly follow the concentration addition model. Important paper for understanding nano structuring events
112	Emulsion	Sorafenib	IL-water nano emulsion	Cholinium Geranate	Enhanced biodistribution and elimination half-life of kinase inhibitor
113	Emulsion	Sorafenib	IL-water nano emulsion	Cholinium Geranate	Continuation of 112, suggesting the IL prevents sorafenib from leaving the cell once internalized
114	Surface Modification	N/A	Graphene	Various choline chloride - carboxylic acid DESs	Improved biocompatibility of graphene by IL functionalization, potential use as a new nanocarrier for small molecules
115	Surface Modification	N/A	Silver/Manganese oxide/bentonite nanocomposites	N-trimethyl-N-propylammonium bis(trifluoromethanesulfonyl)imide	Biocompatible, antibacterial nanocomposites that are synthesized and capped using IL
116	Surface Modification	N/A	Chitosan	Tetramethylammonium hydroxide	Enhanced time stability of chitosan-based microparticles as potential drug carriers
117	Nanocomposites	N/A	montmorillonite layers	4,4′-Bis(2-ethoxymethylimidazolium)azobenzene N-Methylimidazole	UV responsive "molecular jacks" that could be used to create smart sunscreens
118	Responsive Nanocarriers	Doxorubicin	zeolitic imidazolate framework-8	1-Butyl-3- methylimidazolium hexafluorophosphate	Microwave sensitive nanocarriers for externally controllable chemotherapeutic release
119	Responsive Nanocarriers	Doxorubicin	Zirconia/TPP/iRGD nanospheres	1-Butyl-3-methylimidazolium hexafluorophosphate	Microwave-sensitive mitochondria-targeting nanocarriers for externally controllable chemotherapeutic release
120	Surface Modification	Curcumin	Carbon dots	1,3-dibutylimidazolium nitrate and a variety of imidazolium-based ILs	Enhanced cucumin delivery into cells making it a more efficacious cancer therapy
121	Responsive Nanocarriers	Curcumin	silica-graphene oxide nanoparticles	Aspartic acid-based	pH sensitive with potentially widespread pharmaceutical applications

Reference Number	*Nanocarrier Application*	*Therapeutic Payload*	*Nanomaterial*	*Ionic Liquid(s) Used*	*Findings/Application*
122	Surface Modification	N/A	PLGA nanoparticles	Cholinium hexenoate	Protein avoidant nanoparticles with enhanced biodistribution to lungs
123	Nanocomposites	Mefenamic Acid	poly(L-lactide)	Choline mefenamate and di(2-hydroxyethyl)dimethyl ammonium mefenamate	API-IL nanocomposites for carriers of sparingly soluble small molecule drugs
124	Nanocomposites	Mefenamic Acid	poly(L-lactide)	various quaternary ammonium ILs	Same as previous but explores a wider variety of IL structures
125	Responsive Nanocarriers	Methotrexate	montmorillonite layers	1,2,3- benzotriazolium hexafluorophosphate	pH sensitive anti-cancer drug release
126	Responsive Nanocarriers	Doxorubicin	Hyaluronic/Folic/p-hydroxyazobenzene/DMAEMA	1-vinyl-3-hexylimidazolium bis(trifluoromethylsulfonyl)imide	pH and light responsive nanocarriers for externally controllable tumor targeting and release of chemotherapeutics
127	Nanocomposites	Quercetin, Indomethacin, Erythromycin	choline-methacrylate copolymer	choline salicylate and choline sulfacetamide	dual delivery of anti-inflammatory and antibacterial agents by encapsulation of erythromycin in salicylate poly-IL nanoparticles
128	Other	N/A	Platinum/PNIPAM	3-[11-(2-bromo-2-methyl-1-oxopropoxy)undecyl]-1-vinylimidazolium bromide	Nanomotors which have non-Brownian motion in hydrogen peroxide solution
129	Nanocomposites	N/A	PNIPAM	Various choline based ILs	Study on the phase behavior of PNIPAM and IL mixtures
130	Synthesis	Fenbufen	Carbon nanotubes/4-vinylpyridine/ ethylene glycol dimethacrylate	1-butyl-3-methylimidazoliumtetrafluoroborate	Synthesis of nanocomposites for anti-inflammatory drug delivery applications
131	Synthesis	N/A	Bovine Serum Albumin	1-butyl-3-methyl imidazolium tetrafluoroborate	IL allowed for facile-synthesis of BSA nanoparticles
132	Synthesis	N/A	Chlorotoxin/cellulose	N-methylpyrrolidinium hydrogen sulfate	Synthesis of nanoparticles with enhanced glioblastoma uptake in IL solvent
133	Synthesis	Indomethacin	Starch	1-hydroxypropyl-3-methylimidazolium acetate	Starch nanoparticles synthesized using IL-in-oil microemulsion showed better release of model drug than normal starch particles
134	Nanocomposites	Doxorubicin and Methotrexate	Chitosan/Iron Oxide/mPEG	1-methyl-3-(oxiran-2-ylmethyl)-1H-imidazol-3-ium chloride	Targeted "stealth" chitosan-IL nanocomposites for potential breast cancer treatment
135	Responsive Nanocarriers	Pyrene or Ciprofloxacin	IL-water nanoemulsion	[ProC 10][FeCl 3 Br]	Magnetic nanocarrier of hydrophobic drugs

Supporting Information Table 6. Protein Stabilization

Reference Number	*Application/Protein Class*	*Protein/Peptide*	*Ionic Liquid(s) Used*	*Stabilization Improvements (generally time or temperature)*	*Findings/Application*
136	Thermal Stabilization	Cytochrome c	Choline dihydrogen phosphate	Stabilized protein above 90°C	IL helped to stabilize protein against heat degradation
137	Other/Various	Alcohol dehydrogenase	quaternary ammonium (Ammoeng110)	N/A	Purification and stabilization of proteins from cell lysates
138	Thermal Stabilization	Ribonuclease A	Choline dihydrogen phosphate 1-ethyl-3-methylimidazolium dicyanamide	Increased Tm by 20°C at higher concentrations	IL structure has a significant effect on protein stability choline-based increased thermal stability, imidazolium decreased
139	Enzyme Catalysis	α-chymotrypsin	Various ILs from choline, imidazolium, and phosphonium families	N/A	Found that triethyl ammonium acetate can help with refolding
140	Enzyme Catalysis	Lysozyme	ethylammonium nitrate	Recovery of 90% of enzyme after two cycles, compared to 20% in IL-free buffer	IL increased enzyme recovery after multiple cycles with 80% recovery after 10 cycles
141	Interaction Assessment	Bovine Serum Albumin	1-tetradecyl-3-methylimidazolium bromide	N/A	Denaturation of protein appears to be due to the hydrophobic interactions between IL and tryptophan residues
142	Enzyme Catalysis	Various enzymes	Various	N/A	Early review of enzyme stability, activity, and interactions in IL formulations
143	Interaction Assessment	Various cyclic dipeptides	Various ammonium-based ILs	N/A	Showed that ILs stabilize the structure of dipeptides by exclusion from the protein surface
144	Various	Various	Various	N/A	Early review for selective design of ionic liquids in protein systems
145	Therapeutic Proteins	Insulin	various imidazolium and ammonium-based ILs	Recovery of 80% of monomer secondary structure after cryopreservation	Cryopreservation of amyloid fibers with later monomer recovery
146	Interaction Assessment	Xaa-Pro Dipeptides	1- butyl-1-methylpyrrolidinium bis(trifluoromethylsulfonyl) imide	N/A	Ionic liquids can be used to stabilize normally unfavorable dipeptide conformations
147	Enzyme Catalysis	Lysozyme and BSA	1-dodecyl-3-methyl imidazolium bromide ([C12mim]Br)	N/A	Dilational Rheology to asses protein-IL interactions at an oil-water interface
148	Enzyme Catalysis Thermal Stabilization	Lysozyme	1-ethyl-3-methylimidazolium bis-(trifluoromethylsulfonyl)imide and 1-butyl-3-alkylimidazolium bromide	Increase stability and activity limits of lysozyme from 75°C to 120°C	Microemulsions with ethylene glycol core and IL shells for better lysozyme/enzyme shelf life

Reference Number	*Application/Protein Class*	*Protein/Peptide*	*Ionic Liquid(s) Used*	*Stabilization Improvements (generally time or temperature)*	*Findings/Application*
149	Enzyme Catalysis	Lipase	1-Butyl-3-methylimidazolium trifluoromethanesulfonate	N/A	Positively charged amino acids near binding cleft exclude IL cations which help to stabilize enzymes, while hydrophobic amino acids at the same location can cause loss of enzyme function in IL formulations
150	Enzyme Catalysis	Lysozyme	N-alkyl-N-methylmorpholinium bromide	Extended the retention of bacterolytic activity (70% in control vs 90% in IL formulation) after 4 weeks	Extending lysozyme/enzyme shelf life
151	Enzyme Catalysis	Lipase	1-(3-Aminopropyl)-imidazolium hexafluorophosphate	Improved initial enzyme activity by 6.68 times and repeated use activity by about 30%	Enhancing lipase initial and retention of activity
152	Interaction Assessment	ϵ-poly-l-lysine	2-hydroxyethyl ammonium formate	N/A	Formation of a thermoreversible soft gel and molecular docking suggest cation stabilizes the polypeptide
153	Thermal Stabilization	Insulin	cholinium glutamate	Increase denaturation temperature by 9.6°C	Better storage stability at higher ambient temperatures
154	Enzyme Catalysis	Versatile peroxidase	Choline chloride glycerol	about a 4-fold improvement in activity as measured by Michaelis-Menten kinetics	Molecular dynamics showed solvent exposure of hydrophobic residues,
155	Interaction Assessment	Phospholipid bilayers	Various imidazolium and ammonium-based ILs	N/A	Claim that hydrogen bonding plays the most important intramolecular force role in protein-IL stability
156	Interaction Assessment	N/A	N/A	N/A	Review focuses entirely on protein-IL interactions
157	Interaction Assessment	Bovine Serum Albumin	N-2’,3’-epoxypropyl-Nmethyl-2-oxopyrrolidinium chloride	N/A	Molecular docking and spectroscopic experiments elucidate some protein-IL hydrophobic interactions are key
158	Interaction Assessment	Bovine Serum Albumin	N-2’,3’-epoxypropyl-Nmethyl-2-oxopyrrolidinium salicylate	N/A	Molecular docking and spectroscopic experiments elucidate some protein-IL interactions
159	Enzyme Catalysis	Glucose Oxidase	(1-alkyl 3-methyl-imidozolium bromides	N/A	Ionic liquid with more hydrophobic character prevented enzyme activity at similar concentrations
160	Thermal Stabilization	Bovine Serum Albumin	Various choline ILs	Increases thermal stability by 16°C	Assessment of IL-BSA interactions and the thermal stabilization effect

Reference Number	Application/Protein Class	Protein/Peptide	Ionic Liquid(s) Used	Stabilization Improvements (generally time or temperature)	Findings/Application
161	Thermal Stabilization	β-lactoglobulin	Choline-based IL mixtures	N/A	ILs increased thermal stability of β-lactoglobulin
162	Enzyme Catalysis	Lysozyme	1-octyl-3-methylimidazolium dodecylbenzenesulfonate	Enhanced activity by about 10%	IL at concentration around 1 mM enhanced enzyme activity
163	Enzyme Catalysis	cytochrome-*c*	Alginic acid-derived DES	5.5-fold enzyme activity	Alginate-derived DES can be used to enhance enzyme activity drastically
164	Interaction Assessment	Collagen	choline serinate	Increased thermal stability from 61°C to 87°C	Using IL coated nanoparticles can allow for stabilization of fibrous proteins
165	Interaction Assessment	Green Fluorescent Protein	1-butyl-3-methylimidazolium and 1-butyl-1-methylpyrrolidinium cations with chloride, acetate, and triflate anions	N/A	Suggest that multiple analytical techniques (CD, UV/Vis, NMR, SAXS) should be used to properly assess protein stability in ILs
166	Thermal Stabilization	Various	Various	N/A	Review covering how mixtures of ILs can work synergistically to stabilize proteins
167	Various/Other	Various	Various	N/A	Review covering how ILs can play a role in protein refolding *in vitro*
168	Various/Other	Various	Various	N/A	Review of ILs aiding in amyloidogenesis

Supporting Information Table 7. Antimicrobial

Reference Number	Ionic Liquid(s) or ionic liquid class Used	Gram-negative Bacteria	Gram-positive bacteria	Fungi	Mold	Membrane Interaction Assessment
169	methylimidazolium chloride	V. fischeri				
170	1-alkyl-3-methyl imidazolium bromide and 1-alkyl-3-methyl pyridinium bromide ILs	E. coli<br>P. fluorescens	S. aureus<br>B. subtilis	S. cerevisiae		
171	1-alkyl-3- methylimidazolium chloride ILs	E. coli	S. aureus<br>E. faecium<br>E. hirae			
172	1-alkylquinolinium bromide ILs	E. coli<br>P. aeruginosa<br>P. mirabilis<br>K. aerogenes	S. aureus<br>MRSA<br>S. epidermidis<br>MRSE<br>B. cereus	C. tropicalis		

Reference Number	*Ionic Liquid(s) or ionic liquid class Used*	*Gram-negative Bacteria*	*Gram-positive bacteria*	*Fungi*	*Mold*	*Membrane Interaction Assessment*
173	1-alkyl-3-methylimidazolium and 1-alkylpyridinium bromides	E. coli<br>K. pneumoniae<br>P. aeruginosa	S. aureus<br>M. luteus<br>S. epidermidis<br>B. subtilis	C. albicans		
174	Gemini imidazolium	E. coli				
175	$[C_{16}MIm][Cl]$ and $[(C_{10})_2MIm][Cl]$	E. coli	S. aureus	C. albicans<br>C. tropicalis		
176	Anthracene-Imidazolium Bromide	N/A	S. haemolyticus<br>S. epidermidis			
177	theophylline	E. coli	N/A			
178	CAGE	E. coli	N/A			
179	triphenylamine-phosphonium	E. coli<br>K. pneumoniae<br>A. baumannii<br>P. aeruginosa<br>E. aerogenes	S. aureus<br>E. faecium			
180	tetradecyltrimethylammonium theophyllinate	E. coli	B. cereus			
181	2- (methacryloyloxy)ethyl]dimethylheptyl ammonium dodecylbenzenesulfonate	S. maltophilia	M. leteus			
182	imidazolium	E. coli<br>S. Tuphimurium<br>C. freundii<br>P. mirabllis<br>P. aeruginosa<br>A. fischeri	S. aureus<br>L. moncytogenes<br>B. cereus<br>E. faecalis<br>L. sakei<br>L. lactis			
183	[Glycine]Cl	E. coli	S. aureus	M. albicans		

Reference Number	*Ionic Liquid(s) or ionic liquid class Used*	***Gram-negative Bacteria***	*Gram-positive bacteria*	***Fungi***	***Mold***	***Membrane Interaction Assessment***
184	[MIP-tPP][BF_4], [MIP-tPP][PF_6], and [MIP-tPP][CF_3COO]	N/A	S. aureus			
185	1-allyl-3-methylimidazolium chloride	E. coli	S. aureus			
186	salicylidene-iminothiazole and benzylidene-bis-iminothiazole	E. coli<br>A. hydrophilia<br>Vibrio	S. aureus			
187	3-(2- (methacryloyloxy)ethyl)-1-alkyl imidazoliums	E. coli	N/A			
188	1-vinyl-3-butylimidazolium bromide and 1-vinyl-3-butylimidazolium acrylamide	E. coli	S. aureus<br>B. subtilis	C. albicans	Asp. niger<br>Asp. Oryzae<br>Rhizopus	
189	1-butyl-3-methylimidazolium hexafluorophosphate	N/A	S. epidermidis			
190	1-vinyl-3-butylimmidazolium chloride and 1-vinyl-3-ethylimidazolium tetrafluoroborate	E. coli	S. aureus<br>VREF<br>MRSA			
191	imidazolium-ferrocene	E. coli	S. aureus			
192	1-vinyl-3- hexylimidazolium bromide, 1-vinyl-3-allylimidazolium bromide and 1-vinyl-3-butylimidazolium chloride	P. aeruginosa	S. aureus			
193	Imidazolium, Quartanary Ammonium, and DABCO-diium	E. coli	S. aureus			
194	Various (review of poly(ILs) for antimicrobial purposes	Various	Variouos			
195	choline deoxtcholate and choine laurylsarcosinate	E. coli<br>P. aeruginosa	N/A			
196	cholinium, imidazolium, pyridinium	K. pneumoniae	S. aureus<br>B. subtilis			
197	pyrithione	E. coli	L. monocytogenes<br>G. stearothermophilus			

Reference Number	Ionic Liquid(s) or ionic liquid class Used	Gram-negative Bacteria	Gram-positive bacteria	Fungi	Mold	Membrane Interaction Assessment
198	imidazolium, pyridinium	E. coli<br>P. aeruginosa<br>A. baumannii	S. aureus<br>L. monocytogenes<br>B. subtilis<br>S. epidermidis	C. albicans		
199	imidazolium	E. coli<br>K. pneumoniae<br>P. aeruginosa	S. aureus<br>B. subtilis			
200	Pyrrolidinium	E. coli	S. aureus			
201	imidazolium	E. coli	S. aureus			
202	1-butyl-3-methylimidazolium chloride and choline chloride					Neutron refractory assessment of IL-membrane interactions
203	1-butyl-3-methylimidazolium chloride and hexafluorophosphate					Molecular Dynamics of IL-membrane interactions
204	Various imidazolium and choline ILs					Review of IL-membrane interactions
205	diketopyrrolopyrrole-based ionic liquid	E. coli				Molecular dynamics simulations to understand interactions
208	1-butyl-3-methylimidazolium hexafluorophosphate		S. epidermidis			
206	Benzimidazolium based	E. coli	S. aureus<br>MRSA			
207	Choline undeconoate			C. albicans<br>F. solani<br>A. fumigatus		

Supporting Information Table 8. Biosensing

LOD = limit of detection, LLOQ = lower limit of quantification, HLOQ = higher limit of quantification

Reference Number	***Analyte Class***	***Ionic Liquid(s) Used***	***Analyte***	***Immobilized Molecule(s)***	***LOD (g/mL or M)***	***LLOQ (g/mL or M)***	***HLOQ (g/mL or M)***	***Applications/Use***
210	Glucose	1-butyl-3- methylimidazolium hexafluophosphate	Glucose	Glucose oxidase	NS	1.00E-07	2.00E-05	Detection and quantification of blood glucose levels
211	Glucose	1-Butyl-3-methyl-imidazolium tetrafluoroborate	Glucose	Glucose oxidase	1.50E-06	3.00E-06	9.00E-03	Detection and quantification of blood glucose levels
212	Biomarker	1-butyl-3-methylimidazolium hexafluorophosphate	Adrenaline	Laccase	2.93E-07	9.99E-07	2.13E-04	Quantification over wide range for pharmaceutical formulations
213	Biomarker	1-butyl-3-methylimidazolium hexafluorophosphate	Ascorbic Acid<br>Dopamine	Single Wall Carbon Nanotubes	4.00E-13<br>8.00E-14	NS	NS	Detection of dopamine even in presence of ascorbic acid
214	Glucose	n-octylpyridinum hexafluorophosphate	Glucose<br>Ethyl Alcohol	Glucose Oxidase<br>Alcohol Dehydrogenase	NS	NS	NS	Early use of carbon nanotubes in IL-biosensing
215	Therapeutic	1-ethyl-3-methylimidazolium hexafluorophosphate	Diclofenac	$Cu(OH)_2$ nanoparticles	4.00E-08	1.80E-07	1.19E-04	Detection of small molecule drugs in serum samples
216	Glucose	1-(3-Aminopropyl)-3-methylimidazolium bromide	Glucose	Glucose oxidase	NS	2.00E-03	1.60E-02	Glucose detection within the relevant human blood glucose level range
217	Internalized Chemicals	1-Ethyl-3-methylimidazolium tetrafluoroborate	Catechol	Graphene	8.00E-09	2.50E-08	1.11E-05	Functionalization of graphene with IL for biosensor
218	Therapeutic	1- butyl-1-methylpyrrolidinium bis(trifluoromethylsulfonyl)imide	Losartan	Graphene	1.82E-09	3.00E-09	1.00E-02	Detection of antihypertensive drug for treating patients with heart disease

Reference Number	Analyte Class	Ionic Liquid(s) Used	Analyte	Immobilized Molecule(s)	LOD (g/mL or M)	LLOQ (g/mL or M)	HLOQ (g/mL or M)	Applications/Use
219	Biomarker	1-ethyl-3-methylimidazolium bis(trifluoromethylsulfonyl)imide	Dopamine	N/A	3.30E-08	2.00E-07	3.28E-04	Detection of dopamine in urine
220	Internalized Chemicals	1-hexylpyridinium hexafluorophosphate	Tricholoroacetic acid	Myglobin	3.30E-04	1.00E-03	2.00E-01	TCA used widely in topical cosmetic treatments
221	Therapeutic	n-hexyl-3- methylimidazolium hexafluorophosphate	Epinine	N/A	2.00E-07	7.00E-07	9.00E-04	Monitoring serum levels of epinine a catecholamine drug used to treat congestive heart failure
222	Therapeutic	1-butyl-3-methylimidazolium hexafluorophosphate	6-thioguanine<br>6-mercaptopurine<br>azathioprine	ZnO Nanoparticles	5.00E-08<br>1.10E-07<br>9.00E-08	2.00E-07<br>3.00E-07<br>4.00E-07	2.50E-04<br>4.00E-04<br>4.00E-04	Monitoring of serum levels of three common thiopurines which are used for cancer and autoimmune treatment
223	Therapeutic	1-butyl-3-methylimidazolium tetrafluoroborate	Doxorubicin<br>Dasatinib	N/A	9.00E-09<br>5.00E-07	7.00E-08<br>1.00E-06	5.00E-04<br>1.2E-03	Simultaneous detection of these two commonly used chemotherapeutics
224	Therapeutic	1-butyl-1-methylpiperidinium hexafluorophosphate	Daclatasvir<br>Sofosbuvir<br>Ledipasvir	Cobalt Nanoparticles	2.77E-10<br>1.86E-09<br>7.29E-09	2.00E-07<br>7.00E-08<br>3.00E-07	1.00E-06<br>1.00E-06<br>8.00E-06	Detection of hepatitis C antivirals
225	Biomarker	1-butyl-3-methylimidazolium tetrafluoroborate	Nitric Oxide	NO Reductase	7.00E-08	2.30E-07	4.76E-06	NO is an important cell signaling molecule, and has implications in angiogenesis
226	Biomarker	1-butyl-3-methylimidazolium chloride	Cholesterol	Cholesterol Oxidase	4.80E-07	1.00E-06	1.20E-05	
227	Biomarker	1-butyl-3-methylimidazolium tetrafluoroborate	C-reactive Protein	anti-CRP antibodies and BSA	5.00E-12	1.00E-10	1.00E-06	Detection of clinically relevant inflammatory biomarker for cardiovascular diseases.

Reference Number	**Analyte Class**	**Ionic Liquid(s) Used**	**Analyte**	**Immobilized Molecule(s)**	**LOD (g/mL or M)**	**LLOQ (g/mL or M)**	**HLOQ (g/mL or M)**	**Applications/Use**
228	Therapeutic	1-butyl-3-methylimidazolium hexafluorophosphate	Irinotecan	Gold nanoparticles	1.55E-09	4.00E-09	1.79E-06	Detection of chemotherapeutic in presence of electroactive molecules
229	Internalized Chemicals	choline phenylalanine	Ethyl Alcohol	Alcohol Dehydrogenase	7.00E-03	2.20E-02	1.00E-01	Detection of ethyl alcohol in serum samples
230	Glucose	choline phenylalanine	Glucose<br>Ethyl Alcohol	Glucose Oxidase<br>Alcohol Dehydrogenase	3.35E-04<br>3.70E-03	1.01E-03<br>1.11E-02	1.05E-02<br>1.20E-01	Beverage glucose detection<br>of ethyl alcohol in serum samples
231	Biomarker	1-butyl-3-methylimidazolium tetrafluoroborate and choline dihydrogen phosphate	Interleukin 6<br>Cortisol	anti IL-6 antibody<br>antil cortisol antibody	2.00E-13<br>1.00E-10	NS	NS	Detection of biomarkers in human sweat
232	Biomarker	1-dodecyl-3-methylimidazolium bromide	Hydrogen Peroxide	Horseradish Peroxidase	2.50E-07	2.00E-06	4.20E-04	Biological metabolism studies
233	Internalized Chemicals	1-vinyl-3-ethylimidazolium bromide	Catechol	Tyrosinase	2.00E-05	3.90E-05	2.50E-04	
234	Therapeutic	1-butyl-3-methylimidazolium hexafluorophosphate	microRNA-34a	DNA probe	8.20E-07	2.00E-06	1.00E-05	Detection of microRNA in human serum
235	Biomarker	1-Hexylpyridinium hexafluorophosphate	DNA	ssDNA probe	3.17E-14	1.00E-13	1.00E-06	DNA detection
236	Therapeutic	1-octylpyridinium hexaflourophosphate	Doxepin	N/A	2.10E-08	5.00E-08	2.40E-05	Antidepressant sensor for serum and pharmaceutical formulations

Reference Number	*Analyte Class*	*Ionic Liquid(s) Used*	*Analyte*	*Immobilized Molecule(s)*	*LOD (g/mL or M)*	*LLOQ (g/mL or M)*	*HLOQ (g/mL or M)*	*Applications/Use*
237	Biomarker	1-butyl-3-methylimidazolium tetrafluoroborate	Cortisol	anti cortisol antibody	5.00E-09<br>1.00E-08<br>1.00E-09	NS	NS	Detection of cortisol in sweat, urine, and saliva
238	Therapeutic	1-butyl-3-methylimidazolium hexafluorophosphate	Meloxicam	N/A	1.00E-09	5.00E-09	1.50E-06	Ultrasound assisted *in situ* microextraction
239	Therapeutic	1-butyl-3-methylimidazolium hexafluorophosphate<br>1-butyl-3-methylimidazolium tetrafluoroborate	Duloxetine	N/A	8.00E-07	2.00E-06	1.50E-03	Ultrasound assisted *in situ* microextraction
240	Therapeutic	1-vinyl-3-octylimidazolium bromide	Empagliflozin<br>Metformin<br>Canagliflozin	N/A	1.30E-09<br>6.00E-09<br>8.00E-10	5.00E-09<br>2.00E-08<br>5.00E-09	1.20E-06<br>1.80E-06<br>1.00E-07	Extraction of antidiabetic drugs from human plasma
241	Various	Various	Various	N/A	Various	Various	Various	Review of ILs used in extraction of biological samples
242	Internalized Chemicals	1-alkyl-3-methylimidazolium hexafluorophosphate	Ethanol	Alcohol Dehydrogenase	5.00E-06	1.20E-05	2.50E-01	Chemiluminescent biosensor
243	Other/Various	1-(2′-hydroxylethyl)-3-methylimidazolium bis-(trifluoromethane sulfonimide)	p-phenylenediamine	N/A	7.00E-09	2.00E-08	1.00E-05	Detection of a commonly used textile molecule in urine samples
244	Therapeutic	1-hexyl-3-methylimidazolium tryptophan	Cu^{2+}<br>Levodopa	N/A	1.70E-08<br>1.60E-07	5.10E-08<br>5.50E-07	1.60E-05<br>5.00E-06	"on-off" biosensor for dual use detection
245	Other/Various	1-ethyl-3-methylimidazolium bis(trifluoromethylsulfonyl)imide	N/A	N/A	N/A	N/A	N/A	Ultra-sensitive pressure sensor for potential, synthetic skin
